# Integration of Bio-Enzyme-Treated Super-Wood and AIE-Based Nonwoven Fabric for Efficient Evaporating the Wastewater with High Concentration of Ammonia Nitrogen

**DOI:** 10.1007/s40820-025-01685-5

**Published:** 2025-03-10

**Authors:** Qian Ding, Bingqi Jin, Yinxia Zheng, Huiru Zhao, Jun Wang, Haoxuan Li, Dong Wang, Ben Zhong Tang

**Affiliations:** 1 Engineering Research Center of Technical Textiles, Ministry of Education, College of Materials Science and Engineering, Donghua University, Shanghai, 201620 People’s Republic of China; 2 State Key Laboratory of Pollution Control and Resources Reuse, School of Environmental Science and Engineering, Tongji University, Shanghai, 200092 People’s Republic of China; 3 Nonwoven Technology Laboratory, College of Textile Science and Engineering, Jiangnan University, Wuxi, 214122 People’s Republic of China; 4 Centre for AIE Research, Shenzhen Key Laboratory of Polymer Science and Technology, Guangdong Research Center for Interfacial Engineering of Functional Materials, College of Material Science and Engineering, Shenzhen University, Shenzhen, 518061 People’s Republic of China; 5 School of Science and Engineering, Shenzhen Institute of Aggregate Science and Technology, The Chinese University of Hong Kong, Shenzhen (CUHK-Shenzhen), Shenzhen, 518172 People’s Republic of China

**Keywords:** Wood aerogel, AIE, Anti-biofouling, Solar-driven interface evaporation, Ammonia nitrogen wastewater treatment

## Abstract

The treatment of ammonia nitrogen wastewater (ANW) has garnered significant attention due to the ecology, and even biology is under increasing threat from over discharge ANW. Conventional ANW treatment methods often encounter challenges such as complex processes, high costs and secondary pollution. Considerable progress has been made in employing solar-induced evaporators for wastewater treatment. However, there remain notable barriers to transitioning from fundamental research to practical applications, including insufficient evaporation rates and inadequate resistance to biofouling. Herein, we propose a novel evaporator, which comprises a bio-enzyme-treated wood aerogel that serves as water pumping and storage layer, a cost-effective multi-walled carbon nanotubes coated hydrophobic/hydrophilic fibrous nonwoven mat functioning as photothermal evaporation layer, and aggregation-induced emission (AIE) molecules incorporated as anti-biofouling agent. The resultant bioinspired evaporator demonstrates a high evaporation rate of 12.83 kg m^−2^ h^−1^ when treating simulated ANW containing 30 wt% NH_4_Cl under 1.0 sun of illumination. AIE-doped evaporator exhibits remarkable photodynamic antibacterial activity against mildew and bacteria, ensuring outstanding resistance to biofouling over extended periods of wastewater treatment. When enhanced by natural wind under 1.0 sun irradiation, the evaporator achieves an impressive evaporation rate exceeding 20 kg m^−2^ h^−1^. This advancement represents a promising and viable approach for the effective removal of ammonia nitrogen wastewater.

**Supplementary Information:**

The online version contains supplementary material available at 10.1007/s40820-025-01685-5.

## Highlights

- Bio-enzyme-treated super-wood and aggregation-induced emission (AIE)-based nonwoven fabric is integrated into a solar evaporator.
- The evaporator demonstrates a high evaporation rate of 12.83 kg m^−2^ h^−1^ when treating simulated wastewater containing 30 wt% NH_4_Cl under 1.0 sun of illumination.
- AIE-doped evaporator exhibits remarkable photodynamic antibacterial activity against mildew and bacteria.

**Supplementary Information:**

The online version contains supplementary material available at 10.1007/s40820-025-01685-5.

## Graphical abstract

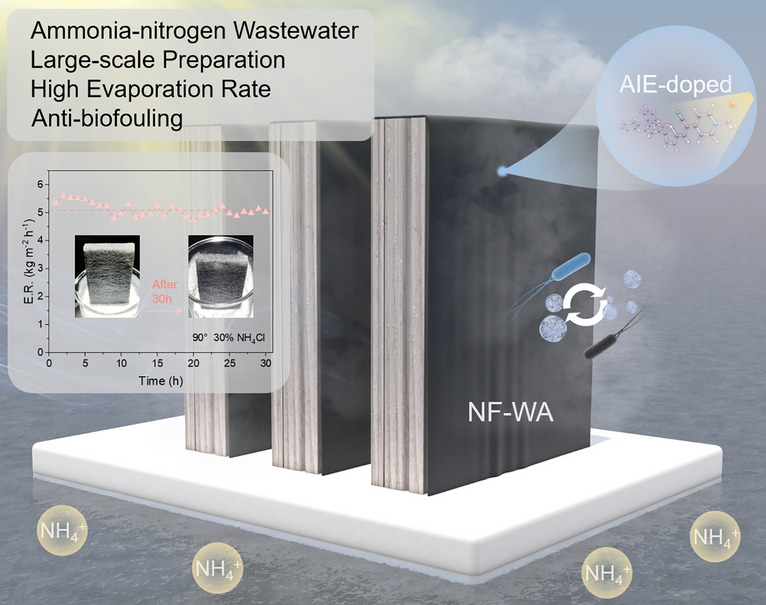

## Introduction

With the development of industry and agriculture, especially in the fields of biopharmaceuticals, fermentation and textile printing and dyeing, the clean water, ecology and even biology are under increasing threat from over discharge of ammonia nitrogen wastewater (ANW) [1–3]. However, the commonly ANW treatment methods primarily consist of gas stripping [4, 5], chemical precipitation [6, 7], biological treatment [8], adsorption [9] and breakpoint chlorination techniques [10], which suffer from low efficiency, high cost and secondary pollution. For example, gas stripping and chemical precipitation methods can effectively treat high concentrations ANW, but the formation of by-products and residues cause secondary pollution [11]. Therefore, it is urgent to explore a novel strategy for separating ammonia nitrogen from wastewater that is characterized by low carbon emission, high effective and eco-friendly.

Solar-driven interfacial evaporation (SIE) technology has emerged as a promising and sustainable method for desalination, garnering considerable attention due to its unique advantages, including the use of inexhaustible solar energy, low costs, and minimal environmental impact [12–16]. Unfortunately, the insufficient research on vapor collection strategies has long been considered a critical limitation of SIE technology, impeding its large-scale implementation and practical applications in desalination [17]. In the context of wastewater treatment, these limitations may be less impactful, as the primary focus is on achieving high evaporation rates and long-term operational stability, rather than vapor collection [18–20]. This makes SIE technology particularly suitable for treating wastewater, including ANW. Nevertheless, several challenges persist in the practical application of SIE, such as difficulty maintaining high evaporation caused by salt deposition and insufficient water supply, poor long-term stability due to weak anti-biofouling capability and difficult to scale application because of complex manufacturing techniques [21, 22]. Thus, great efforts are still required to innovate evaporator with high evaporation performance, superior stability, and easy to scale up.

In recent decades, various materials together with novel structure have been developed for SIE system [23, 24]. Among these, fiber-based evaporator offers incomparable advantages, including low-cost, interconnected pore structure and large-scale preparation, but inevitably possesses weak capacity of wicking, transferring and storing water. Conversely, alkali-treated wood demonstrates excellent water transportation and storage, but is limited by unregulated interfacial wettability and low evaporation rate and weak antibacterial. Therefore, combining bio-enzyme-treated wood with fiber-based evaporator has the potential to enhance evaporation performance. To further improve anti-biofouling properties and ensure long-term stability, aggregation-induced emission (AIE) materials, as emerging photosensitizers, can perfectly couple photothermal conversion with the generation of reactive oxygen species (ROS) via rational design, making them as an ideal alternative for tailoring evaporator with superior anti-biofouling performance [25–27].

**Fig. 1:**
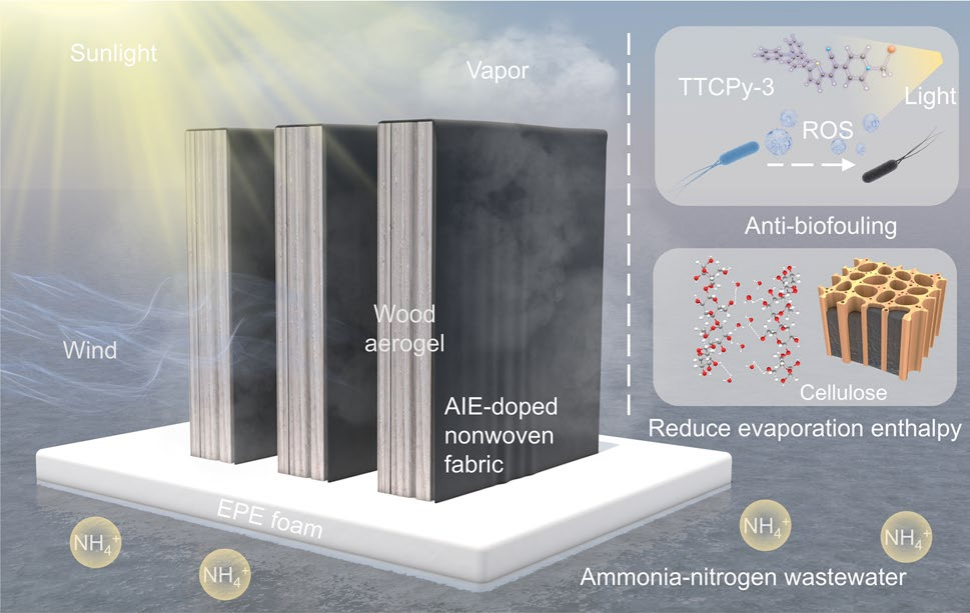
Design of solar wastewater evaporator and a schematic diagram of natural transpiration inspired nonwoven fabric and wood aerogel evaporator (NF-WA) system, exhibiting anti-biofouling and high-flux purification capacities for practical application. The NF-WA consists of two main parts, including the AIE-doped nonwoven fabric and wood aerogel featuring a hierarchical porous structure

Herein, we present a novel evaporator inspired by natural plant transpiration, which integrates a bio-enzyme-treated wood aerogel (WA) as a water pumping and storage layer, a hydrophobic/hydrophilic fiber-based nonwoven fabric (NF) as the evaporation layer, the low-cost multi-walled carbon nanotubes (MWCNTs) photothermal conversion agent and AIE molecules as the anti-biofouling layer. This innovative design (referred to as the nonwoven fabric and wood aerogel, NF-WA) synergistically combines these components to achieve a remarkably high evaporation rate and efficient ROS generation, facilitating effective anti-biofouling effects simultaneously (Fig. 1). Owing to its sufficient water supply, low evaporation enthalpy, interconnected pore structure and excellent photodynamic properties, our evaporator exhibited an excellent purification efficiency of > 99.9% for wastewater containing 30 wt% ammonia nitrogen. In addition, the multifunctional evaporator shows excellent long-term stability and robust outdoor evaporation performance. Notably, an impressively higher evaporation rate over 20 kg m^−2^ h^−1^ is obtained with the assistance of the wind under 1.0 sun irradiation, providing a feasible and cost-effective solution for the treatment of ANW. Besides, this study also explores an environmentally friendly method for fabricating delignified balsa wood via bio-enzyme treatment under mild conditions, offering a potential alternative to traditional high-temperature alkali treatment processes.

## Experimental Section

### Materials and Reagents

The wood-softening enzyme was obtained from Shanghai Kangdien Biotechnology Co. Ltd, China. Hydrogen peroxide (30 % H_2_O_2_) were purchased from Sinopharm Chemical Reagent Co. Ltd., China. AIE materials (TTCPy-3) were provided from Prof. Peihong Xiao. Multi-wall carbon nanotubes (MWCNTs, > 97% of purity) with outer diameter ranging 40–60 nm and length less than 2 μm were supplied by Shenzhen Nanotech Port Co. Ltd., China. A dispersant for carbon nanotubes aqueous solution (XFZ20) was purchased from Nanjing XFNANO Materials Tech Co. Ltd., China. Deionized (DI) water was obtained from an ultra-pure water system (CM-RO-C2). All the balsa wood used in this work was purchased from taobao.com, while EPE foam was purchased from local market. PE/PP ES fiber and viscose fiber were provided from Zhejiang Anshun Pettechs Fibre Co. Ltd., China, and Hangzhou Youbiao Technology Co. Ltd, China, respectively.

### Fabrication of Nonwoven Fabric and Wood Aerogels Evaporator (NF-WA)

Firstly, 0.25 g carbon nanotube water dispersant was added to 0.1 L DI water and sonicated 0.5 h. Then, 1.0 g multi-wall carbon nanotubes (MWCNTs) were mixed into the dispersion solution under the sonication. The final concentration of MWCNTs in the dispersion was 10.0 g L^−1^. Subsequently, a centrifugation process (2000 r min^−1^, 20 min) was utilized to obtain a uniform supernatant of carbon nanotubes. Finally, a coating solution with photothermal performance was prepared by adding 0.1 g L^−1^ of AIE (TTCPy-3) powder and sonicating for 1.0 h.

In typical experimental procedures, hydrophobic PP/PE core–shell structure fibers and viscose fibers were processed into webs using a carding machine. Needle punching, a widely-used nonwoven bonding technique, involved the vertical insertion of felt needles into the fibers, producing punctures that facilitated intertwining. The AIE-doped photothermal coating was subsequently applied to the surface of the nonwoven fabric via a spraying method. The shell layer of the core–shell structure was then melted using hot air, serving as a binder to secure the photothermal coating. The preparation of wood aerogels (WA) from natural balsa wood involved enzyme treatment followed by a freeze-drying process. Initially, wood slices were immersed in boiling DI water for 5 min and then placed in a 0.4 g L^−1^ aqueous solution of wood enzyme at 45 °C for one week. The wood slices were subsequently treated in boiling 2.5 mol L^−1^ H_2_O_2_ for 1 h, until they achieved a white appearance. Finally, the WA was produced via freeze-drying for 24 h. The as-obtained nonwoven fabric was initially partitioned into different shapes. The WA surface and the NF were assembled by a facile suspension method.

### Characterization of NF-WA Evaporator

The morphology of the balsa wood, wood aerogel, nonwoven fabric and microbes was characterized by a field emission scanning electron microscope (FE-SEM, Hitachi, SU8010). Water vapor transmission (WVT) rate was tested by water vapor transmittance testing system (Labthink, W3-060). The water contact angle characterization images of the bilayer fabric and hydrophilic copper needle were recorded based on a surface contact angle meter (KRÜSS Scientific, DSA25) by dropping 10 µL DI water on the surface. The pore size of the nonwoven fabric was measured by a capillary flow porometer (PMI, CFP-1100A). The thermography characterization was performed by an infrared camera (FLIR E8). The absorption spectra of the solar absorber were calculated based on the reflectance and transmission spectra measured by a UV–Vis–NIR Spectrometer Lambda 950 equipped with an integrating sphere. The concentrations of NH_4_^+^ ion in simulated wastewater and purified water were examined by ion chromatography (Thermo Scientific Aquion). The absorption spectrum of simulated dye wastewater and purified water was tested using a UV–Vis spectrophotometer (Shimadzu UV-2600). The chemical composition was comprehensively determined by Fourier-transform infrared spectrometer (FTIR, Nicolet is10, Thermo fisher). Differential scanning calorimetry (METTLER TOLEDO, DSC 3 +) measurements were used for measuring the vaporization energy of pure water and water in NF-WA. Thermal conductivities of balsa wood, wood aerogel and nonwoven fabric were measured by heat flow method thermal conductivity analyzer (NETZSCH HFM446). Mechanical properties were measured by the universal material testing system (HST, WDW-1). Zeta potential was tested by multi-angle particle size and high sensitivity Zeta potential analyzer (Brookhaven, NanoBrook 90plus PALS).

### Molecular Dynamics (MD) Simulations

MD simulations were conducted to compare the evaporation of pure water and the water on NF-WA at 313 K. All MD simulations were performed using Amorphous Cell and Forcite modules in Material Studio 2020. In order to simplify the model, we ignore the role of the photothermal components MWCNTs and TTCPy-3 in the process of water evaporation due to its relatively small content and the phase of materials are not considered. The lattice dimensions are 4.7 nm × 3.5 nm × 11.4 nm in x, y, and z directions. The chain model was constructed and optimized using 18 propylene molecules (PP) and 8 cellulose molecules (cellulose). From the 3D perspective, the cellulose and PP segments are arranged alternately.

**Fig. 2:**
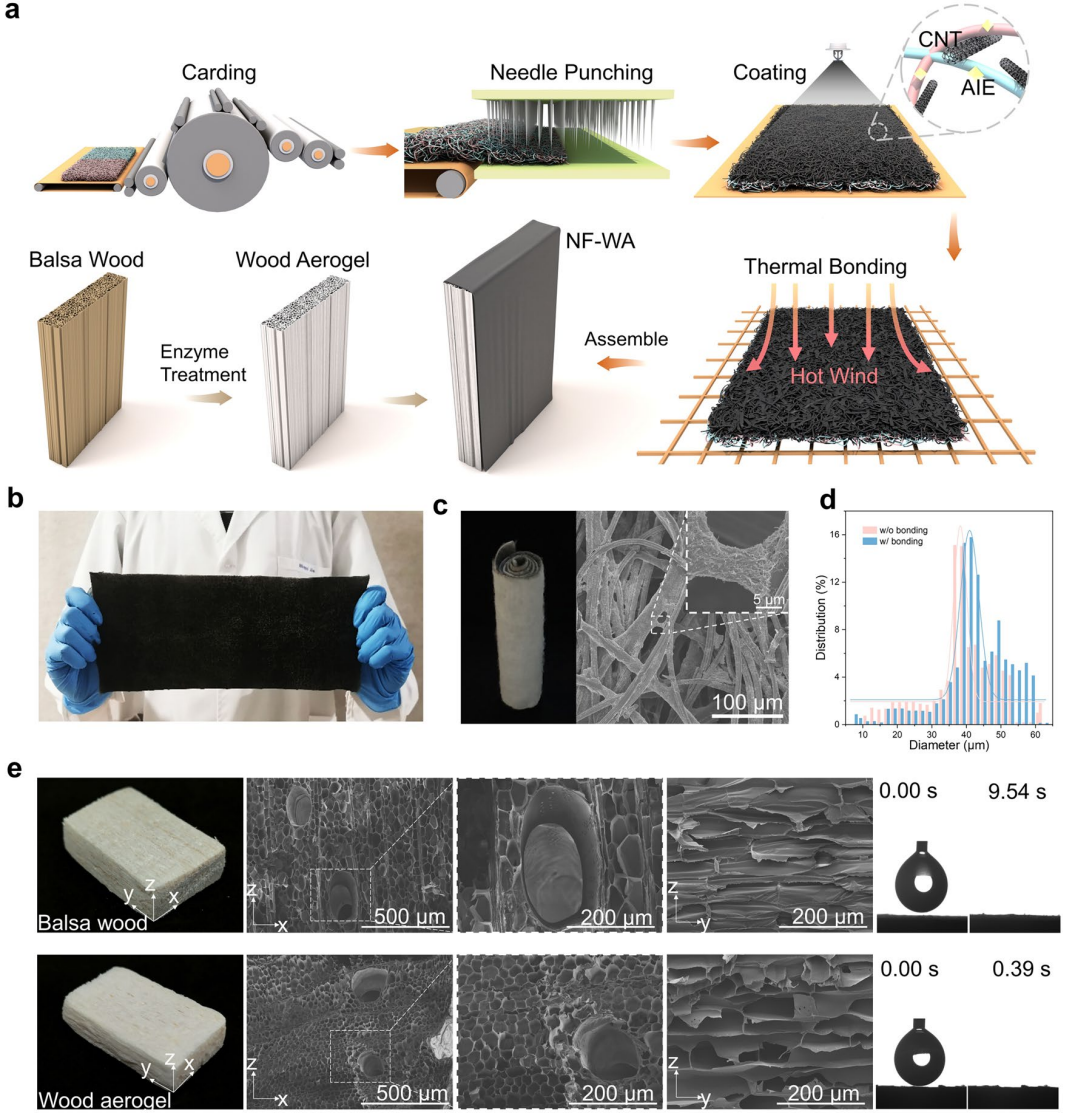
**a** Process flow diagram for the NF-WA integration. **b** Photograph of AIE-doped nonwoven fabric with photothermal effect. **c** Optical and SEM images of AIE-doped photothermal conversion nonwoven fabric after roll forming. **d** Pore size distributions of the nonwoven fabric with/without thermal bonding. **e** Photographs of balsa wood and wood aerogel, as well as the microscopic structure of both materials as observed by SEM from different directions and magnifications, are presented alongside corresponding water contact angle diagrams

Besides, a free water layer (1000 water molecules, density: 1.0 g cm^−3^, sectional size: 4.7 nm × 3.5 nm × 1.6 nm) is placed on the surface. For comparison, the similar free water layer (1000 water molecules, density: 1.0 g cm^−3^, size: 4.7 nm × 3.5 nm × 2.2 nm) is also placed on the surface of the bulk water layer with 1000 water molecules (density: 1.0 g cm^−3^, sectional size: 4.7 nm × 3.5 nm). During the simulation, we have fixed the positions of cellulose, PP and water molecules in the bulk water layer, and all the water molecules in free water layers are movable. Then, the molecular structure was performed through a 100 ps NVT simulation on evaporation system to reach the real density and obtain equilibrium structure.

**Fig. 3:**
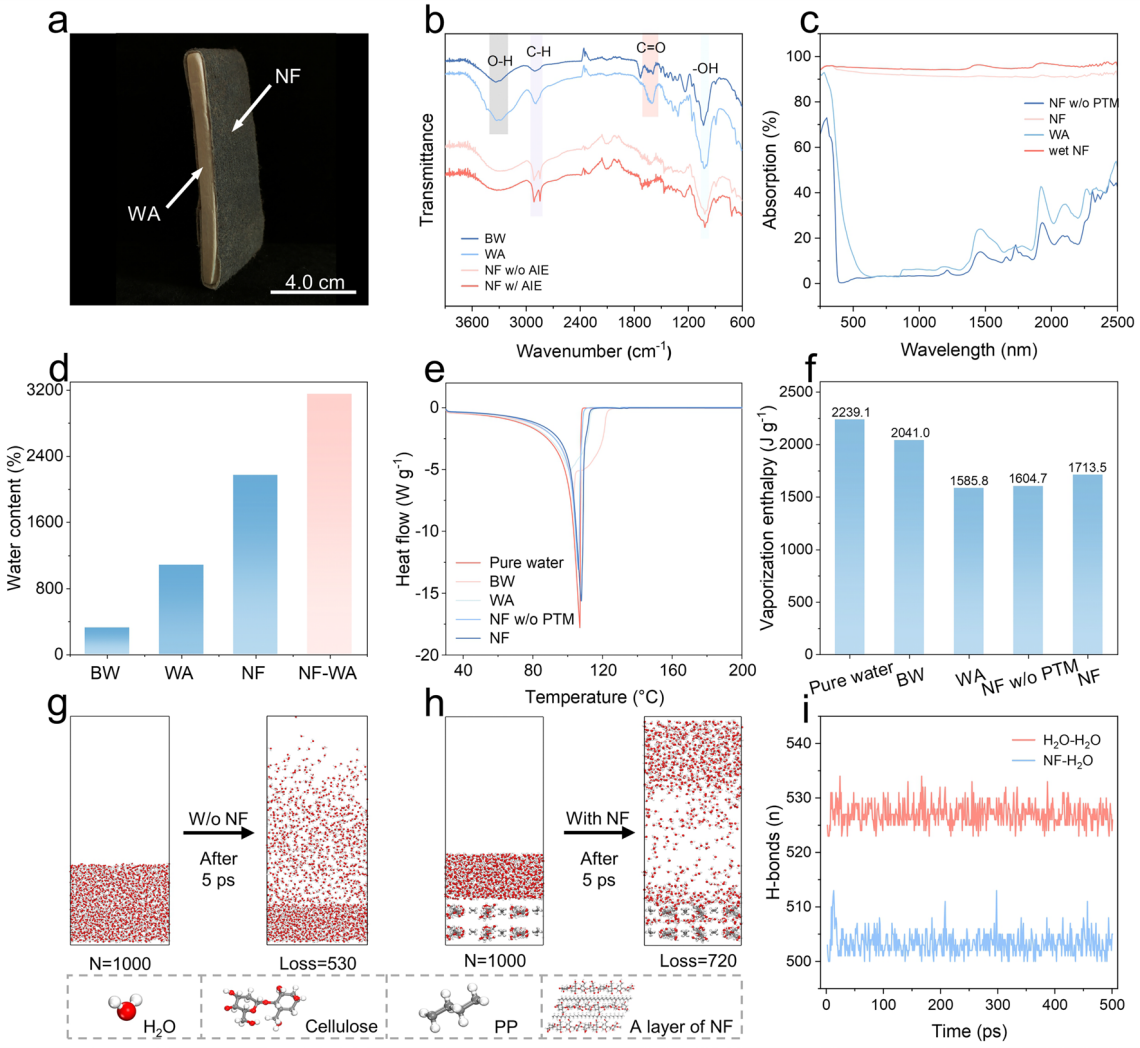
**a** Photograph of the NF-WA. **b** FTIR, and **c** absorbance spectrum of each component of the NF-WA. **d** Water content of the NF-WA. **e** DSC measurement of pure water and water in the NF, and WA. **f** Estimation of the evaporation enthalpy of pure water and water in the Balsa wood (BW), WA and NF. **g** Molecular dynamic simulation of evaporation process: side view of water molecules in the absence of the NF system and **h** in the presence of the NF system.** i** Number of hydrogen bonds of H_2_O-H_2_O and NF-H_2_O system

The interactions between atoms were determined using Universal force field. Nose thermostat was used to control the temperature for NVT MD simulation. The escaped water molecules increase very fast at the beginning to reach evaporation balance. At water–water interface, the escaped water molecule number increases quickly to 530 at 5 ps, and then, it elevates to 627 from 5 to 100 ps with a relatively uniform speed. Importantly, at water-NF-WA interface, the escaped water molecule number is 720 at 5 ps and then ascends to 890 from 5 to 100 ps, which also shows a relatively low but uniform slope. These results indicate that the evaporation rate of water on the NF-WA surface is significantly faster than that of bulk water, attributable to altered hydrogen bonding structures at the various interfaces.

### Indoor and Outdoor Evaporation Performance of NF-WA

Indoor solar evaporation experiments were constructed and tested to assess the performance of the NF-WA under representative laboratory conditions, with a temperature of ~ 30 °C and relative humidity of ~ 60%. The CME-sol8050 simulated sunlight (Microenergy Technology Co., Ltd, China) equipped with an optical filter for the standard AM 1.5G spectrum was provided by a solar simulator. The mass of the water evaporation was recorded by an NV622 electronic balance (OHAUS, USA).

Outdoor experiment was conducted on the roof of the college in Jiangnan University (Wuxi, China). Wind velocity, ambient temperature and solar intensities were recorded by an SW-6036 anemometer (SEWVY, China), TA612C K-type thermocouple (TASI, China) and TES132 solar power meter (TES, Taiwan, China), respectively.

### Antibacterial Activity Assay

75.0 mg NF-WA samples were cut up and placed it into a 6-well plate. Add 0.5 mL of a 1 × 10^6^ CFU mL^−1^ solution of *E. coli* and *S. aureus* to the sample. Next, add 7 mL of a phosphate buffer saline (PBS) solution into the 6-well plate. *E. coli* and *S. aureus* were then treated with or without NF-WA under dark conditions and simulated sunlight for 30 min. Finally, 0.1 mL of bacterial solution was diffused onto agar plates and incubated at 37 °C for 24 h. Colonies were counted, and experiments were performed with two replicates. 0.1 mL (1 × 10^6^ CFU mL^−1^) *E. coli* and *S. aureus* bacteria suspension was pipetted to NF-WA samples on 6-well plates. Bacteria were subjected to treatment with or without NF-WA under both dark conditions and simulated sunlight irradiation for a duration of 30 minutes. Samples were fixed with 2.5% glutaraldehyde and dehydrated by 30%, 50%, 70%, 90%, 95% and 100% ethanol for analysis by field emission scanning electron microscopy (FE-SEM).

## Results and Discussion

### Preparation and Characterization of NF-WA

As illustrated in Fig. 2a, the NF-WA evaporator is fabricated through two key steps: (1) utilizing needle punching and coating techniques to prepare NF with superior photothermal conversion and photodynamic capacities and (2) exploring wood-softening enzymes to treat balsa wood, resulting in a WA with controllable pore sizes. These two elements are then combined to form the NF-WA evaporator. Herein, traditional textile manufacturing technologies are employed for NF fabrication, facilitating easy of scale-up and quality control (Fig. 2b). In addition, the intrinsic advantages of fiber-based NF, such as their large specific surface area and interconnected pore structure, endow the evaporator with efficient water transport, vapor escape as well as low thermal conductivity characteristics. To further enhance the evaporation performance, a hydrophobic/hydrophilic hybrid evaporation interface is constructed by controlling the ratio of hydrophobic (PP/PE ES bicomponent fiber) and hydrophilic (viscose fiber) fibers during the carding process. Following needle punching, the treatment of multi-walled carbon nanotubes (MWCNTs) coating and thermal bonding significantly enhances the mechanical properties of the NF, as evidenced by an increased in strength from 1.1 to 9.6 MPa when compared to the untreated fabric. This improvement suggests enhanced stability and extended service life for water transport and operational durability (Fig. S1). The observed enhancement is likely due to the high-temperature treatment (130 °C), which fuses the polyethylene phase of the bicomponent fibers, thereby reducing fiber slippage, while the polypropylene and viscose fibers, which have higher melting points, retain their original morphology (Fig. 2c). The internal morphological structure of the NF was examined using FE-SEM, revealing that the presence of distinct pinholes is advantageous for vapor escape, while the groove structures of the viscose fibers facilitate enhanced water transport through capillary action (Fig. S2). Importantly, the pore size distributions of the NF, both with and without thermal bonding treatment, exhibit similar patterns, ranging from 35 to 50 μm, indicating that the thermal treatment does not significantly alter the NF structure (Fig. 2d). Thermal bonding also accelerates the soaking time of water as compared to nonwoven fabric without thermal bonding, leading to enhanced water transport efficiency (Fig. S3a, b). Besides, Moreover, the NF demonstrates sufficient durability to withstand intense sonication in liquids, with no significant loss of photothermal materials observed after treatment (Fig. S3c), confirming the stability of the coating on the NF. This observation aligns with previous studies, which suggest that the robust attachment is due to interactions established between the MWCNTs and the polyethylene phase during the thermal bonding process [28].

Balsa wood serves as the framework for the solar evaporator due to its natural hydrophilicity and interconnected pore structure and ensures efficient water transport and low thermal conductivity. To further optimize the hydrophilicity and pore size of the balsa wood, enzyme treatment and freeze-drying methods were employed to generate the WA. Figure 2e presents photographs and SEM images showing the balsa wood before and after bio-enzyme treatment. The treatment partially removes lignin and hemicellulose, resulting in a pure white wood aerogel. The internal interconnected porous structure of balsa wood features well-aligned tracheids (pore diameter 30–100 μm) and vessel channels (pore diameter: 150–300 μm), which are pivotal for rapid water transport along the axial growth direction of the plant. Additionally, there are many micropits (pore diameter: 1–6 μm) distributed on the vessel wall, which can be used to transport water in the radial direction. After enzymatic delignification, the well-aligned tracheids transition to a rough honeycomb structure, which reduces pore size and increases pore density per unit area, enhancing water capillarity [29]. However, there is a minor effect on the lamellar layers of the wood aerogel in the radial direction, which is essential for water transport and diffusion of residual ammonia nitrogen. To assess the hydrophilicity of the enzyme-treated wood aerogel, 10 μL water was dropped on its surface, where it was completely absorbed within a short period of 0.39 s. In contrast, the same volume droplet took 9.54 s to permeate natural balsa wood. This discrepancy indicates that the wood aerogel possesses superior wettability due to the reduced hydrophobicity of lignin, ultimately enhancing the evaporator’s water capillarity [30]. The weight of the WA, after the removal of lignin and hemicellulose, is approximately two-thirds that of natural balsa wood, rendering it ultralight and suitable for placement on bristlegrass (Fig. S4a). In addition, the thermal conductivity of the dry WA and wet WA was 0.067 and 0.163 W m^−1^ K^−1^, respectively, indicating a slight variation in thermal conductivity as compared to natural balsa wood (Fig. S4b). Notably, the thermal conductivity of the NF was measured at 0.029 W m^−1^ K^−1^, which is comparable to that of air, thus improving thermal localization capability.

As shown in Fig. 3a, the AIE-doped nonwoven fabric was tightly wrapped around the wood aerogel, resulting in the successfully fabrication of the NF-WA. Details information regarding the NF-WA is presented in Table. S1. Fourier-transform infrared (FTIR) spectroscopy was used to analyze the internal organic functional groups of NF-WA (Fig. 3b). The peaks observed in the NF-WA, located at 3100 to 3500 and 2800 to 3000 cm^−1^, which correspond to OH groups stretching and C-H stretching, exhibited significant increases. This enhancement indicates an improvement in the hydrophilicity of the NF-WA. The UV–Vis–NIR spectrometer equipped with an integrating sphere was used to evaluate the light absorption. The NF-WA, in both its dry and wet states, demonstrated superior solar absorption capability, exhibiting broad band absorbance of about 94.2% across the solar spectrum from 250 to 2500 nm (Fig. 3c). The temperature of the NF-WA in dry state increased to 80.2 °C within 15 min of 1 sun irradiation, showing the excellent thermal localization capabilities and photothermal conversion properties of the NF-WA (Fig. S5). Because of the specific structure characteristic of wood aerogel through partial removal of hemicellulose, the NF-WA represented an excellent water wicking capacity. As confirmed in Fig. S6, red ink was rapidly drawn to a height of 5 cm by capillary force within 1 min and the NF-WA became completely saturated within 30 min. Therefore, NF-WA exhibits a high affinity for water owing to its excellent hydrophilicity, making it an efficient device for solar interface evaporation. In addition, the hydrophilicity of NF and interconnected pore structure of WA contribute the NF-WA functioning as a high-flux “water tower,” capable of absorbing water equivalent to 32 times its weight (Fig. 3d).

Differential scanning calorimetry (DSC) was used to evaluate the enthalpy of evaporation. The results indicate that both the NF and WA exhibit lower enthalpy values of water evaporation, providing strong evidence of the reduction of the energy barrier associated with water evaporation in the NF-WA structure (Fig. 3e, f). To further investigate the mechanism behind the reduction of evaporation enthalpy in NF-WA systems, MD simulations were conducted to analyze the evaporation of water molecules in the presence and absence of the NF. The crystal structure of each component of NF-WA was generated using the cellulose and polyethylene molecular simulation parameters [31, 32]. A total of 1000 water molecules were placed on the surface of blended cellulose and polyolefin chains, with water molecules being trapped around the cellulose chains through hydrogen bonding. After evaporation for 5 ps at 313.15 K and 101 kPa, 720 water molecules escaped from the surface of the NF, whereas only 530 water molecules evaporated from the bulk water (Fig. 3g, h). This demonstrates that the introduction of amphiphilic NF at the molecular level leads to increased vaporization of water molecules within the NF-WA system. Additionally, the number of hydrogen bonds (H-bonds) in both the bulk water system and the NF system were calculated (Fig. 3i). The results revealed that the number of H-bonds in NF system was lower than that in the bulk water system, suggesting that the NF weakens the crosslinking of H-bonds, thus reducing the enthalpy of evaporation and enhancing overall evaporation performance.

### Solar-Induced Water Evaporation Performance of the NF-WA

**Fig. 4:**
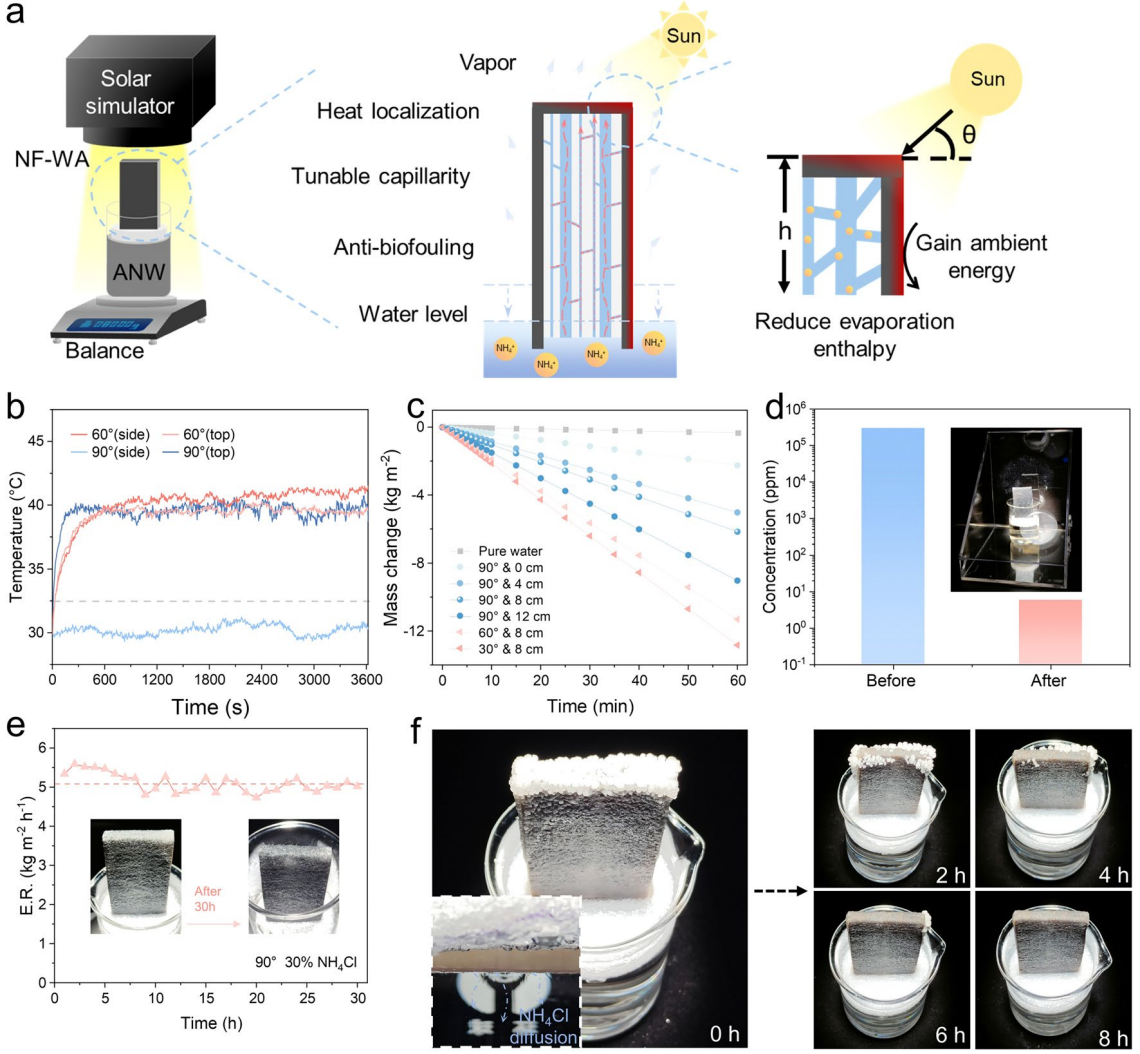
**a** Schematic illustration of NF-WA evaporator under laboratory conditions and working principles under different solar irradiation angles and evaporator heights. **b** Surface temperature variation of the NF-WA at different solar angles under 1.0 sun irradiation.** c** Mass changes of water using pure water and NF-WA under 1.0 sun of irradiation. **d** Concentrations of NH_4_^+^ ions in the simulated ammonia nitrogen wastewater and the collected purified water after vapor condensation. **e** Duration test of NF-WA in 30 wt% NH_4_Cl solution under 1.0 sun irradiation over 30 h period. **f** Time-elapse snapshots show salt rejection from the surface of NF-WA under 1.0 sun irradiation, where sufficient NH_4_Cl salts were placed on the top surface to evaluate salt resistance

To investigate the solar steam generation capacity, the NF-WA evaporator was inserted in polyethylene foam and floated on a beaker filled with simulated wastewater containing 30 wt% NH_4_Cl (Fig. 4a). The angle of sunlight irradiation (θ = 90° and 60°) and the height of the evaporator (h = 0, 4, 8 and 12 cm) significantly affected the evaporation rate, which in turn influenced the flux of wastewater treatment. Upon exposure to the 1 sun irradiation, we monitored the temperature of the NF-WA evaporator using an infrared camera (Fig. S7). As sunlight was directed vertically onto the evaporator (θ = 90°), the surface temperature of the NF-WA evaporator reached 40 °C within 20 min, while the temperature in the side areas remained notably lower than the ambient temperature, demonstrating a heat localization effect and the feasibility of harvesting energy from the environment [33, 34]. However, as the sunlight irradiation angle was adjusted from 90° to 60°, the surface and side temperatures of the evaporator increased to 39.8 and 41.1 °C, respectively, within 1 h (Fig. 4b). As a result, both the surface and sides of the evaporator contributed to the heating area for water evaporation, thereby enhancing the overall evaporation efficiency. The average water evaporation rates recorded were 2.26, 5.03, 6.17 and 9.02 kg m^−2^ h^−1^ at height of 0, 4, 8 and 12 cm, under the vertical irradiation of 1 sun, respectively (Fig. 4c). The evaporation efficiency of solar to vapor generation can be found in our previous work and calculated by the following equations [35, 36]:

1 $$\begin{array}{c}\eta =\frac{\Delta m\times {h}_{lv}}{{C}_{opt}\times {P}_{0}}\end{array}$$

2 $${h}_{lv}=1.91846\times {10}^{6}\times (\frac{T}{T-33.91})$$

where *Δm* is the net evaporation rate. *h*_lv_ denotes the total enthalpy of liquid–vapor change, *C*_opt_ denotes the optical concentration, and *P*_0_ is the solar irradiation at the power density of 1 kW m^–2^. *T* is the temperature (K) of evaporation. After substituting values and converting units, evaporation efficiency of our NF-WA (8 cm, 90°, 1.0 sun) was 296.25%. By optimizing the irradiation angle (θ = 30°) and the height (h = 8 cm) of the NF-WA evaporator, the highest water evaporation rate reached up to 12.83 kg m^−2^ h^−1^, which is approximately 40 times greater than the evaporation rate of pure water. The corresponding evaporation efficiency (η) for the NF-WA evaporator was approximately 761.71% under 1.0 sun irradiation (Fig. S8a). In addition, the evaporation rate of the evaporator further increased to 20.5 kg m^−2^ h^−1^ with a convective flow of 1.9 m s^−1^ under 1.0 sun tilted irradiation, enabling efficient high-flux wastewater purification (Fig. S8b). Compared to the currently reported solar-induced interfacial evaporator, our NF-WA evaporator exhibits the considerable high evaporation rate under 1.0 sun of irradiation, demonstrating the stable and efficient high-flux purification performance (Fig. S9 and Table S2) [25, 37–59]. By analyzing the collected vapor during evaporating wastewater contained 30% of NH_4_Cl, the pH of the collected water is 7.4, which further indicates that the ammonium salt does not decompose during the evaporation process (Fig. S10).

To evaluate the ecological impacts of volatile ammonia during the interface evaporation process of the ANW, we compared ammonia nitrogen wastewater and the purified water collected by evaporation. The results showed that the concentration of NH_4_^+^ in the freshwater was significantly lower than that in the wastewater (Fig. 4d), suggesting that negligible harmful volatile ammonia was released during the interfacial evaporation process. This phenomenon may be attributed to the evaporation interface temperature, which was approximately 50 °C, insufficient to decompose ammonium salts. As shown in Fig. 4e, after 30 h of irradiation, the NF-WA evaporator still maintained an evaporation rate of approximately 5.1 kg m^−2^ h^−1^, demonstrating stable and efficient high-flux purification performance. This performance is primarily due to its inherent three-dimensional interconnected porous structure and amphiphilic hybrid composition. In addition, despite the ultra-high evaporation rate of 5.1 kg m^−2^ h^−1^ and the high concentration of NH_4_Cl (30%) in the simulated wastewater, no salt crystal was observed on the surface of the evaporator after 30 h of irradiation, indicating excellent anti-salt accumulation properties. As illustrated in Fig. 4f, about 20 g of NH_4_Cl salts were directly placed on the top surface of NF-WA, and these added salts gradually dissolved within 8 h under the irradiation of 1.0 sun, affirming the exceptional salt crystals diffusion backflow capacity of the NF-WA. Due to the synergistic effect of NF and WA in water transport, and WA as the predominant mass transfer channel. The inset of Fig. 4f displays the diffusion and convection traces of the NH_4_Cl salts from NF-WA to the underlying bulk water. Taken together, the instinctive porous structure of the fibrous nonwoven mat provides interconnected channels for continuous water transport and vapor release, endowing the NF-WA evaporator with remarkable anti-salt deposition property under long-term solar illumination.

### Anti-Biofouling Capacities of the NF-WA

**Fig. 5:**
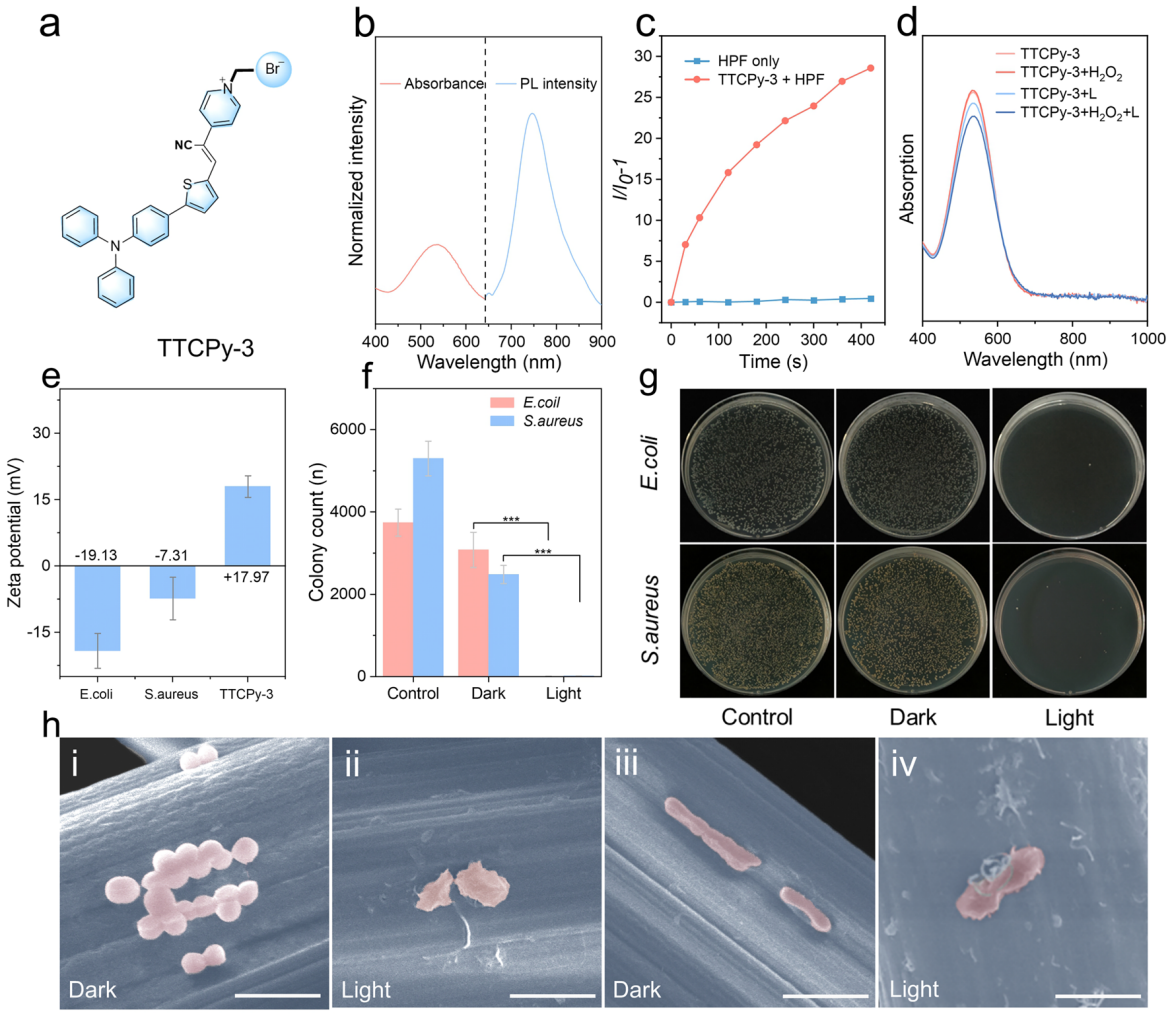
**a** Molecular structure of designed TTCPy-3. **b** Normalized absorption and PL spectra of TTCPy-3 in the DMSO solution and solid state, respectively. **c** Decomposition rates of HPF for ·OH detection in the presence of TTCPy-3 (1 μM) upon white light irradiation (30 mW cm^−2^) for different times. **d** Absorption spectra of TTCPy-3 in DMSO solution with/without light irradiation for 24 h, and with/without the addition of 30% H_2_O_2_ solution. **e** Zeta potential of *E. coli*, *S. aureus* and AIE-doped NF-WA. **f** Colony counts of *S. aureus* and *E. coli* treated with different experimental materials. **g** Agar plates exhibit the bacterial inactivation performance in darkness and light for 30 min. **h** (i, ii) FE-SEM images of *S. aureus* and (iii, iv) *E. coli* cultured on the NF-WA in darkness or upon 1.0 sun irradiation. Scale bar, 2 μm

In addition to anti-salt accumulation, anti-biofouling measures are crucial for ensuring long-term stability in solar-driven evaporation systems. Due to the presence of numerous microorganisms in wastewater, these can attach to the evaporator, forming biofilms after prolonged exposure, which ultimately leads to channel plugging [60]. This issue is particularly pronounced in cellulose-based evaporators, where biofouling adversely affects both internal and surface structures, diminishing the stability and lifespan of the evaporators [61]. Incorporating a rising photosensitizer with AIE characteristics plays a vital role in mitigating biological deposition on evaporators, owing to their superior photodynamic antibacterial properties [62–64]. Therefore, TTCPy-3, a typical donor–acceptor (D-A) structure with strong intramolecular charge transfer (ICT) characteristic, was utilized to impart the NF-WA evaporator with the capacity to generate reactive oxygen species (ROS) to inhibit microorganism proliferation (Fig. 5a) [65]. The maximum absorptions of TTCPy-3 in dimethyl sulfoxide (DMSO) were located at 534 nm and the maximum emission in the solid-state peaking at 746 nm (Fig. 5b). Such large Stokes shift is likely attributable to the anin-π^+^ interactions between bromide (Br^−^) and positive charged pyridine rings [66]. Subsequently, hydroxyphenyl fluorescein (HPF) was employed as indicator to detected ·OH generation of the TTCPy-3, revealing a significantly by 27-fold increase in emission intensity, which demonstrates high efficiency in type I ROS generation (Fig. 5c). Moreover, 9,10-anthracenediyl-bis(methylene) dimalonic acid (ABDA) was used as an indicator for singlet oxygen (^1^O_2_), the absorption intensity of ABDA treated with TTCPy-3 decreased by approximately 3.36%, indicating insufficient ^1^O_2_ generation capacity (Fig. S11a). In contrast, the intensity of dihydrorhodamine 123 (DHR 123) in TTCPy-3 increased more than 346 times after 7 min of white light irradiation, proving a significantly high generation efficiency of a superoxide anion radical $({\text{O}^{-}_{2}})$ by TTCPy-3 (Fig. S11b). Taken together, the TTCPy-3 was capable of generating ROS effectively through type-I mechanism. In addition, the stability of TTCPy-3 against to ROS was assessed. There were no significant changes observed in the absorption spectra of the TTCPy-3 after 24 h of light exposure or upon the addition of H_2_O_2_ (Figs. 5d and S12a, b). However, the absorption peak of the commercially available photosensitizer indocyanine green (ICG) decreased by approximately 63.9% and 62.8% following light irradiation and H_2_O_2_ exposure, respectively, over the same duration. This underscores the superior stability of TTCPy-3 relative to ICG (Fig. S12c). As shown in Fig. 5e, the Zeta potentials of *Escherichia coli* (*E. coli*), *Staphylococcus aureus* (*S. aureus*) and TTCPy-3 suspensions were measured at −19.13, −7.31 and 17.97 mV, respectively. These results indicate that the TTCPy-3 suspensions engaged in electrostatic interactions with both bacteria species, facilitating bacterial eradication through surface adsorption followed by a photodynamic anti-biofouling mechanism [67].

After spraying the TTCPy-3 to the NF, green fluorescence was observed under 365 nm ultraviolet light irradiation, confirming the successful doping of TTCPy-3 on the surface of the NF (Fig. S13). Subsequently, the anti-biofouling properties of the NF-WA against *E. coli* and *S. aureus* were conducted using the plate count method. Within 30 min of sunlight irradiation, 99.67% of *S. aureus* and 99.84% of *E. coli* were killed by NF-WA, indicating that the TTCPy-3 endows the NF-WA evaporator with excellent photodynamic antibacterial properties and effectively inhibits bacteria proliferation both on the surface and within the evaporators (Fig. 5f, g). In comparison, TTCPy-3 doped NF-WA exhibited markedly enhanced photodynamic antibacterial performance relative to the NF-WA without TTCPy-3 (Fig. S14a). To further explore the photodynamic anti-biofouling activity of TTCPy-3, morphological changes of bacteria incubated on the NF-WA in dark and under solar irradiation were obtained by FE-SEM (Fig. 5h). The cellular destruction and surface wrinkle of the *E. coli* and *S. aureus* were observed on the surface of the viscose fiber within 30 min of solar irradiation. In contrast, under dark conditions, both bacterial species exhibited rod and spherical shapes with smooth contours. This observation suggests that the mechanism for bacterial inactivation involves the disruption of cell membranes. The anti-biofouling activity of TTCPy-3 primarily results from the inactivation of biological macromolecules by free radicals generated through the Type-I ROS under solar irradiation [68, 69]. Additionally, the anti-biofouling performance under practical conditions was also evaluated by floating the NF-WA on the simulated wastewater for 72 h. A significant amount of bacterial plaque was observed on the surface of natural balsa wood, likely due to its cellulose structure in a wet state, which provides conducive conditions for bacterial growth (Fig. S14b). However, no mildew accumulation was observed on the surface of the NF-WA (Fig. S14c), further indicating its excellent anti-biofouling capacity.

### Outdoor Waste Water Treatment Performance of the NF-WA-Based Devices

**Fig. 6:**
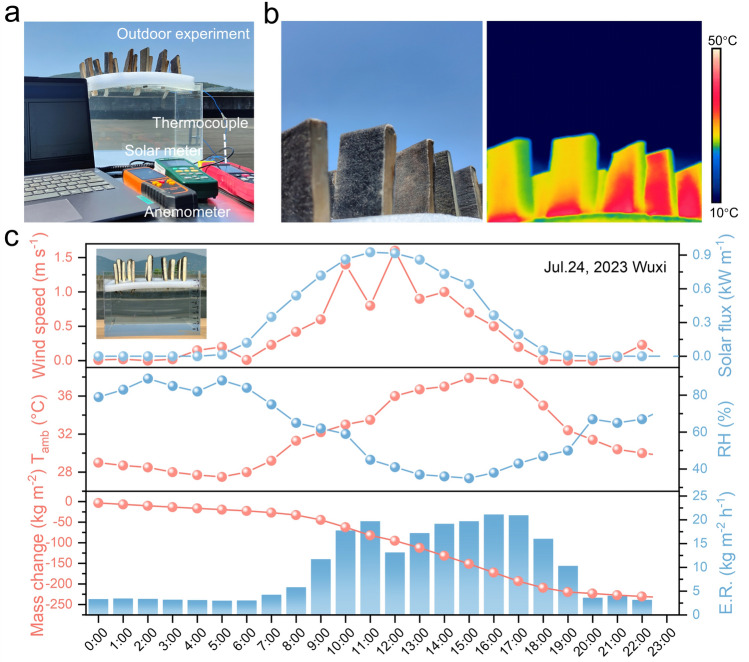
**a** Diagram of the outdoor evaporation experiment utilizing the NF-WA array on the simulated wastewater system. **b** Optical image of the assembled evaporator array floating on the wastewater tank prototype and its corresponding IR image under natural solar irradiation. **c** Measurements of wind speed, solar intensity, ambient temperature (T_amb_), relative humidity (RH%), mass change of the wastewater and evaporation rate (E.R.) of NF-WA during the experiment on July 24, 2023, at Jiangnan University

The widespread adoption of solar interface evaporation technology continues to face significant challenges hindering its advancement [70]. On July 24, 2023, we conducted a comprehensive outdoor solar wastewater purification test utilizing our high-performance solar evaporator atop the roof of Jiangnan University in Wuxi, Jiangsu Province, China (Fig. 6a). As shown in Fig. 6b, the IR image taken at 10:00 AM reveals that the surface temperature of the NF-WA evaporators increased to 31.9 °C, indicating the superior photothermal conversion of the NF-WA. An array of instruments, including a solar meter, anemometer, thermocouple and hygrometer, were used to monitor environmental parameters, such as solar flux, wind speed, ambient temperature and relative humidity (RH). As shown in Fig. 6c, the natural solar flux gradually increased throughout the morning, reached a maximum of 0.9 sun at midday and subsequently declined in the afternoon. The maximum evaporation rate, measured at 21.1 kg m^−2^ h^−1^ for an effective area of 4.6 × 10^–3^ m^2^, occurs at 4:00 PM. This high evaporation efficiency is attributed to both the position of the sun and the angle of irradiation, as well as the peak ambient temperature coupled with the lowest RH experienced in the afternoon, which significantly enhances the rate of solar evaporation [71]. Under natural sunlight exposure for 8 h, the volume of simulated wastewater decreased from 7.1 to 6.6 L, achieving a total evaporation of 0.5 L (Fig. S15). Notably, even during extended periods of darkness, the evaporators maintained an evaporation rate of 3.6 kg m^−2^ h^−1^, indicating their viability for practical application in wastewater treatment.

## Conclusion

In summary, we have demonstrated an innovative solar evaporator inspired by the natural plant transpiration, aiming to achieve efficient wastewater purification and anti-biofouling performance. The evaporator effectively controls capillary action and functions as a highly efficient water transfer channel by optimizing the pore structure of the wood aerogel. We have successfully achieved a high evaporation rate of 12.83 kg m^−2^ h^−1^ under 1.0 sun irradiation. Furthermore, the incorporation of AIE molecules into the NF-WA has endowed it with exceptional photodynamic antibacterial activity against mildew and bacteria. This advancement ensures excellent anti-biofouling performance during long-term wastewater treatment. Notably, we recorded an even higher evaporation rate exceeding 20.0 kg m^−2^ h^−1^ with the assistance of air convection under 1.0 sun irradiation, thereby offering a viable strategy for the removal of ammonia nitrogen wastewater.

## Supplementary Information

Below is the link to the electronic supplementary material.

Supplementary file1 (DOCX 3475 KB)
