# Supplementary material for: Integration of Bio-Enzyme-Treated Super-Wood and AIE-Based Nonwoven Fabric for Efficient Evaporating the Wastewater with High Concentration of Ammonia Nitrogen
Source: Nanomicro Lett. 2025 Mar 10;17:176. doi: 10.1007/s40820-025-01685-5 (PMC11893956; doi:10.1007/s40820-025-01685-5)
Supplement: Supplementary file 1 — Supplementary file1 (DOCX 3475 KB) [file 40820_2025_1685_MOESM1_ESM.docx]

Supporting Information for

**Integration of Bio-Enzyme Treated Super-Wood and AIE Based Nonwoven Fabric for Efficient Evaporating the Wastewater with High Concentration of Ammonia Nitrogen**

Qian Ding^1,+^, Bingqi Jin^2,+^, Yinxia Zheng^3^, Huiru Zhao^3^, Jun Wang^1^, Haoxuan Li^1,^*, Dong Wang^4,^*, Ben Zhong Tang^5,^*

^1^Engineering Research Center of Technical Textiles, Ministry of Education, College of Materials Science and Engineering, Donghua University, Shanghai 201620, P. R. China

^2^State Key Laboratory of Pollution Control and Resources Reuse, School of Environmental Science and Engineering, Tongji University, Shanghai 200092, P. R. China

^3^Nonwoven Technology Laboratory, College of Textile Science and Engineering, Jiangnan University, Wuxi 214122, P. R. China

^4^Centre for AIE Research, Shenzhen Key Laboratory of Polymer Science and Technology, Guangdong Research Center for Interfacial Engineering of Functional Materials, College of Material Science and Engineering, Shenzhen University, Shenzhen 518061, P. R. China

^5^School of Science and Engineering, Shenzhen Institute of Aggregate Science and Technology, The Chinese University of Hong Kong, Shenzhen (CUHK-Shenzhen), Shenzhen 518172, P. R. China

^+^ Qian Ding and Bingqi Jin contributed equally to this work.

*Corresponding authors. E-mail: [hxli@dhu.edu.cn](mailto:hxli@dhu.edu.cn) (Haoxuan Li), [wangd@szu.edu.cn](mailto:wangd@szu.edu.cn) (Dong Wang), [tangbenz@cuhk.edu.cn](mailto:tangbenz@cuhk.edu.cn) (Ben Zhong Tang)

**Supplementary Figures**


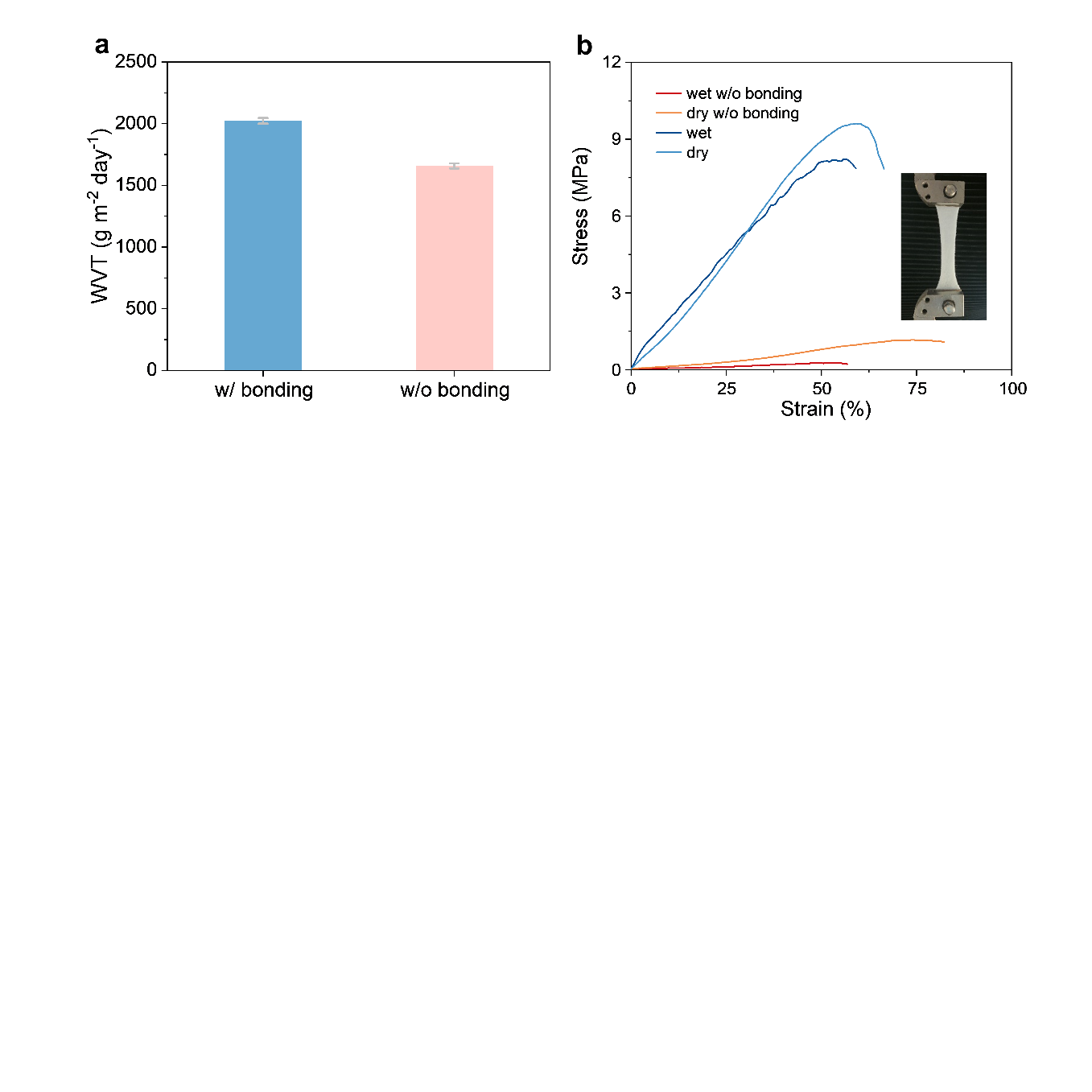


**Fig. S1** (**a**) Water vapor transmission rates (WVT) of the nonwoven fabric with(w/) or without (w/o) thermal bonding. (**b**) Tensile stress-strain curves of the different wetting states nonwoven fabric with or without thermal bonding


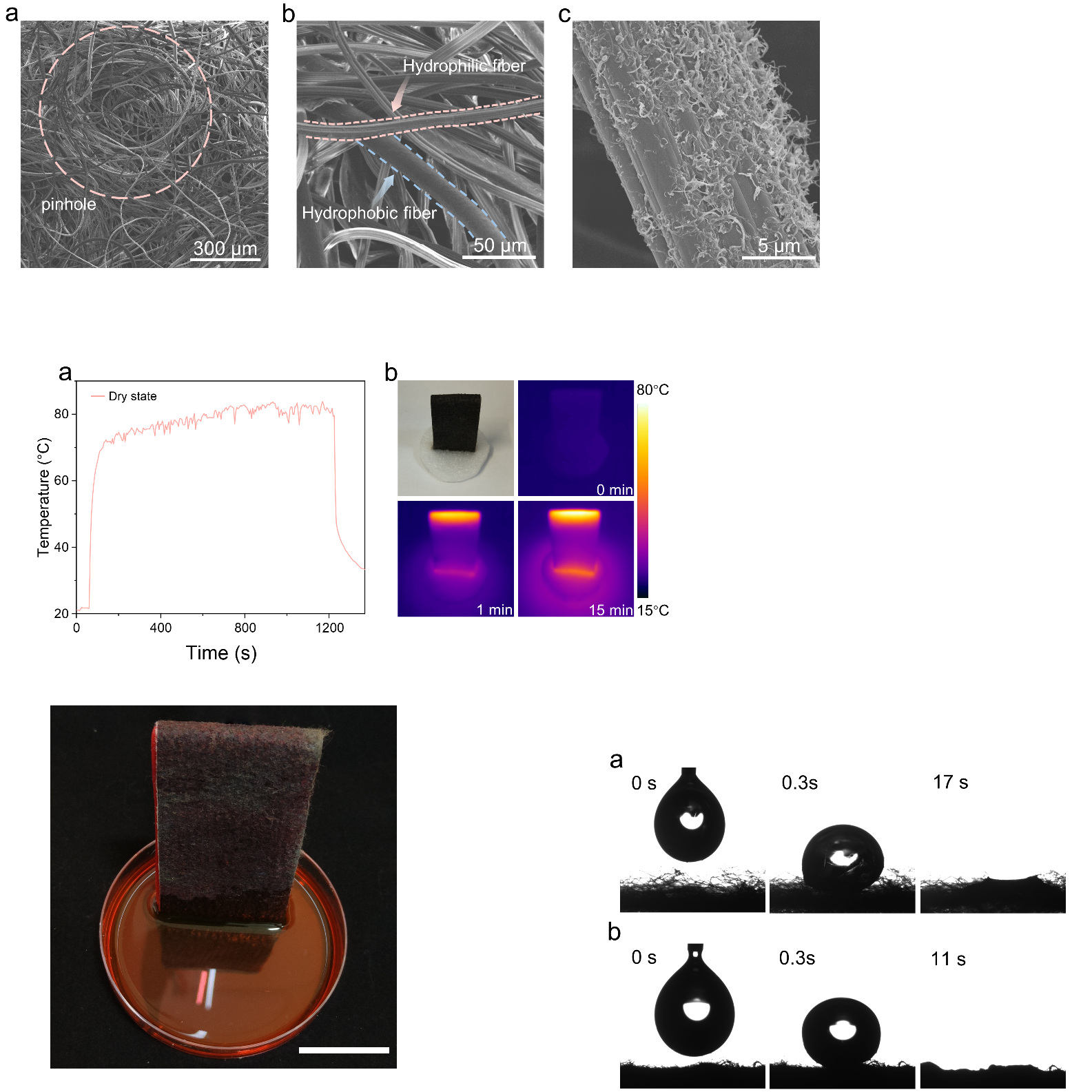


**Fig. S2** (**a**) FE-SEM images of the nonwoven fabric, (**b**) hydrophobic and hydrophilic

hybrid structure and (**c**) photothermal materials coated fiber


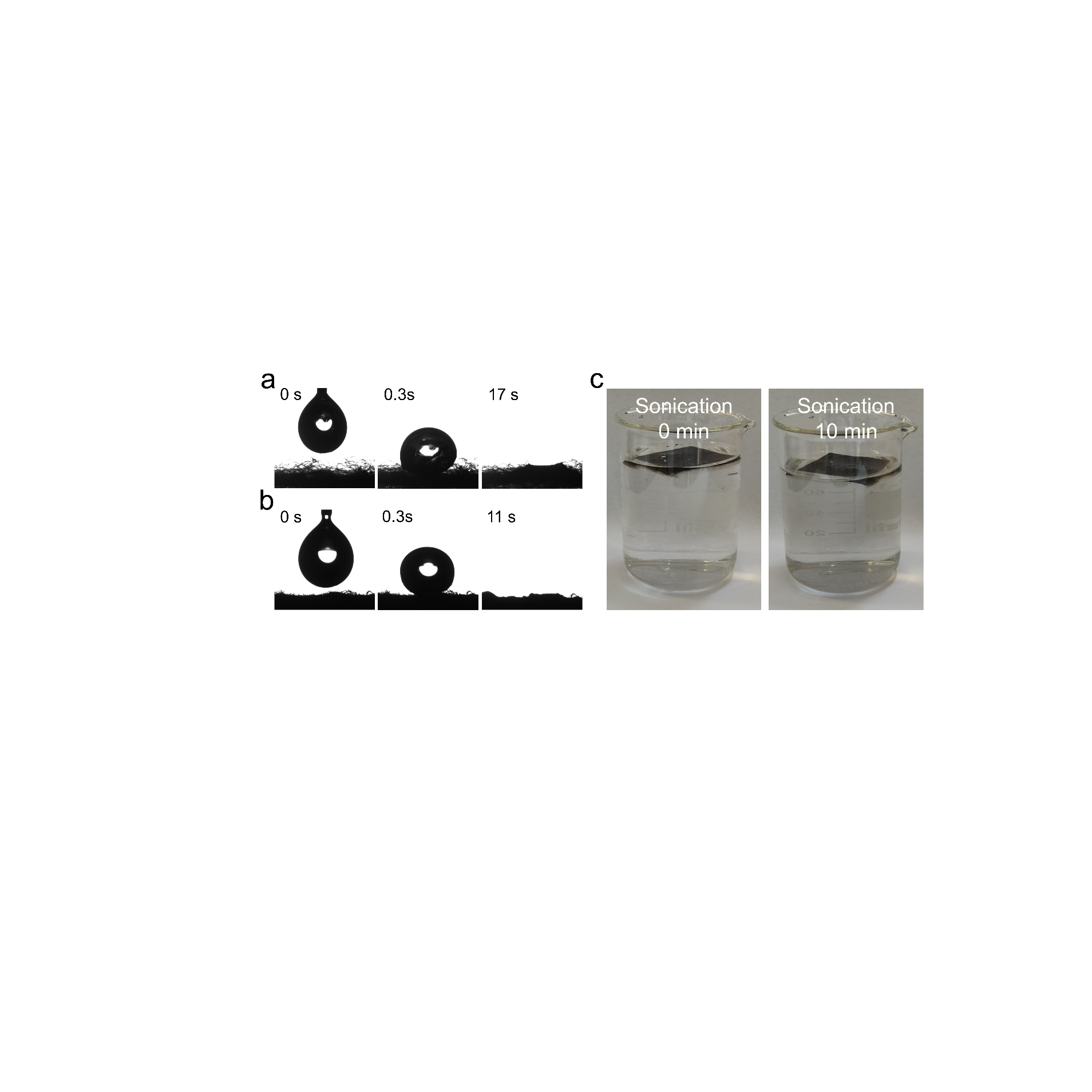


**Fig. S3** (**a**) Water contact angle images of nonwoven fabric without thermal bonding and (**b**) nonwoven fabric with thermal bonding when a water droplet was dropped on their surfaces. (**c**) Digital photograph showing the stability of the photothermal materials against a cycle of 10 min sonication


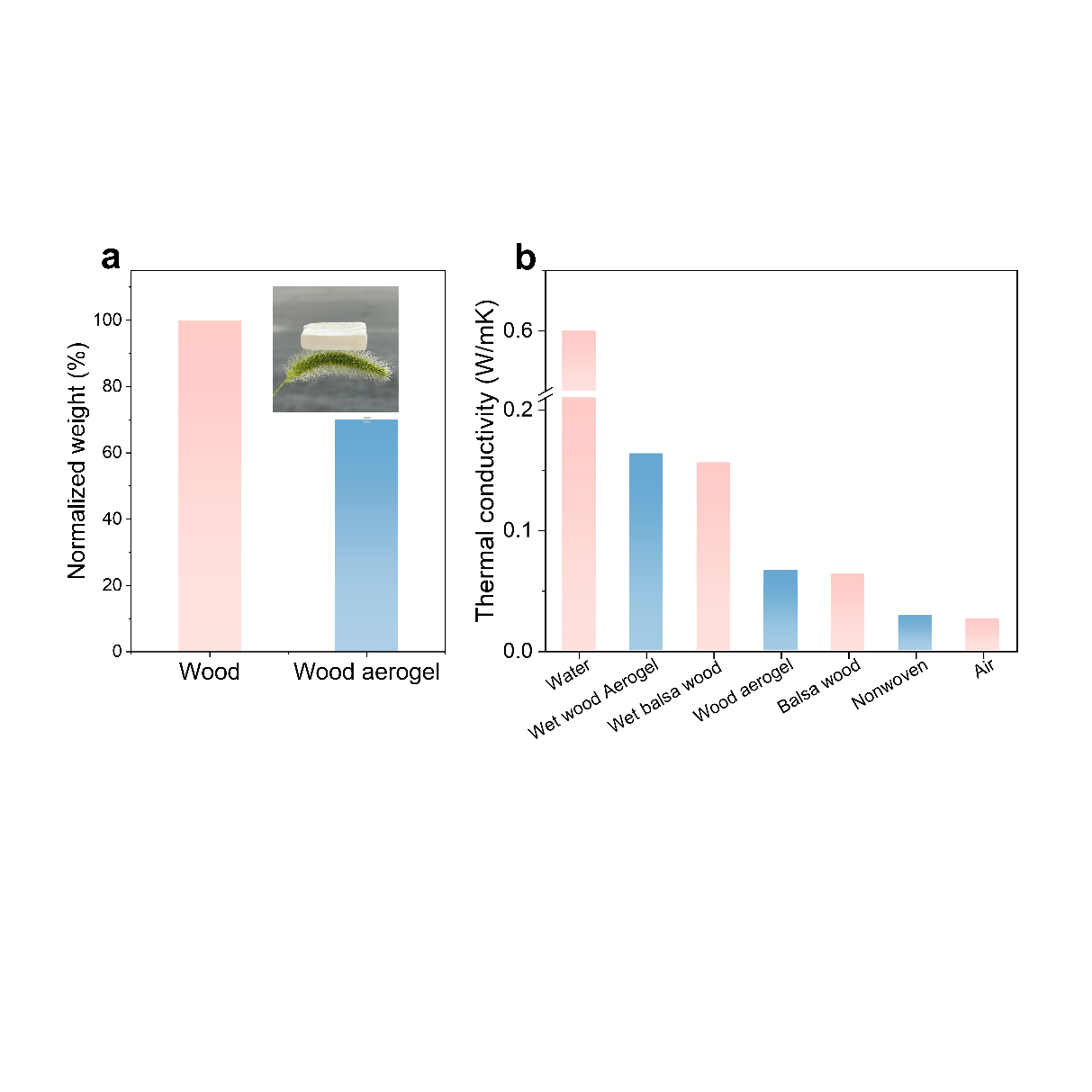


**Fig. S4** (**a**) Weight of balsa wood and the wood aerogel in the dry state. Inset: wood aerogel was placed on a grass like dog's tail (Setaira viridis (L.) Beauv). (**b**) Thermal conductivity of the balsa wood, wet wood aerogel, nonwoven fabric and some common thermal-insulated materials


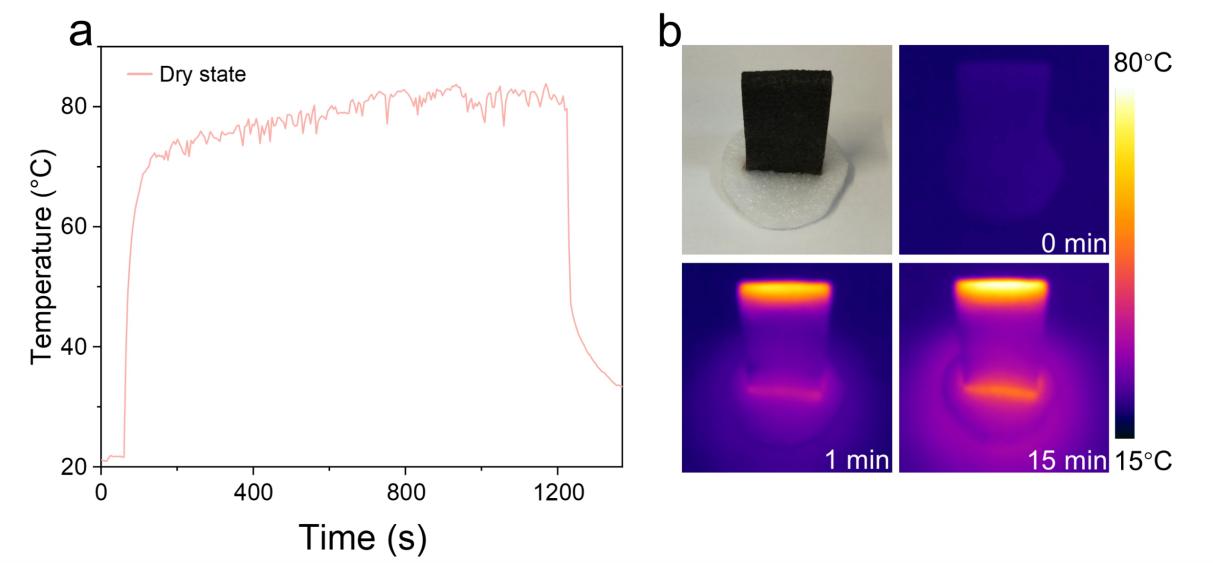


**Fig. S5** (**a**) Temperature change of dry NF-WA surface under 1.0 sun irradiation. (**b**) Optical and IR images of the NF-WA under perpendicular 1.0 sun irradiation


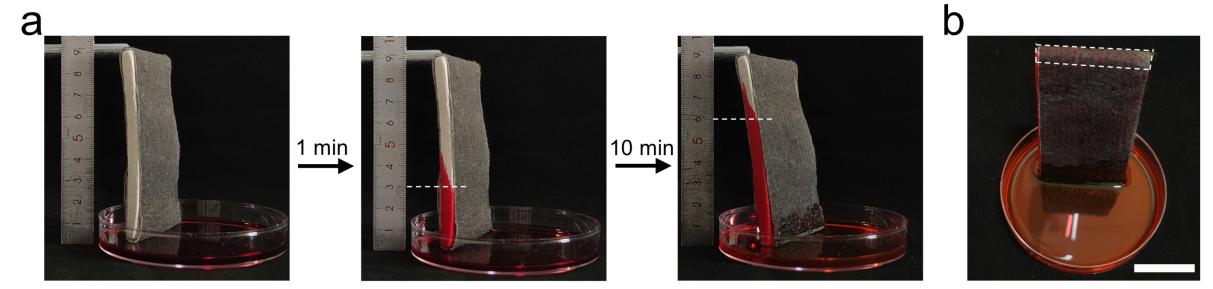


**Fig. S6** (**a**) Optical photos of wicking capacity of NF-WA. (red ink is used to improve the discrimination of the experiment). (**b**) Optical image of NF-WA was totally saturated by the red ink within 30 min. Scale bar, 2 cm


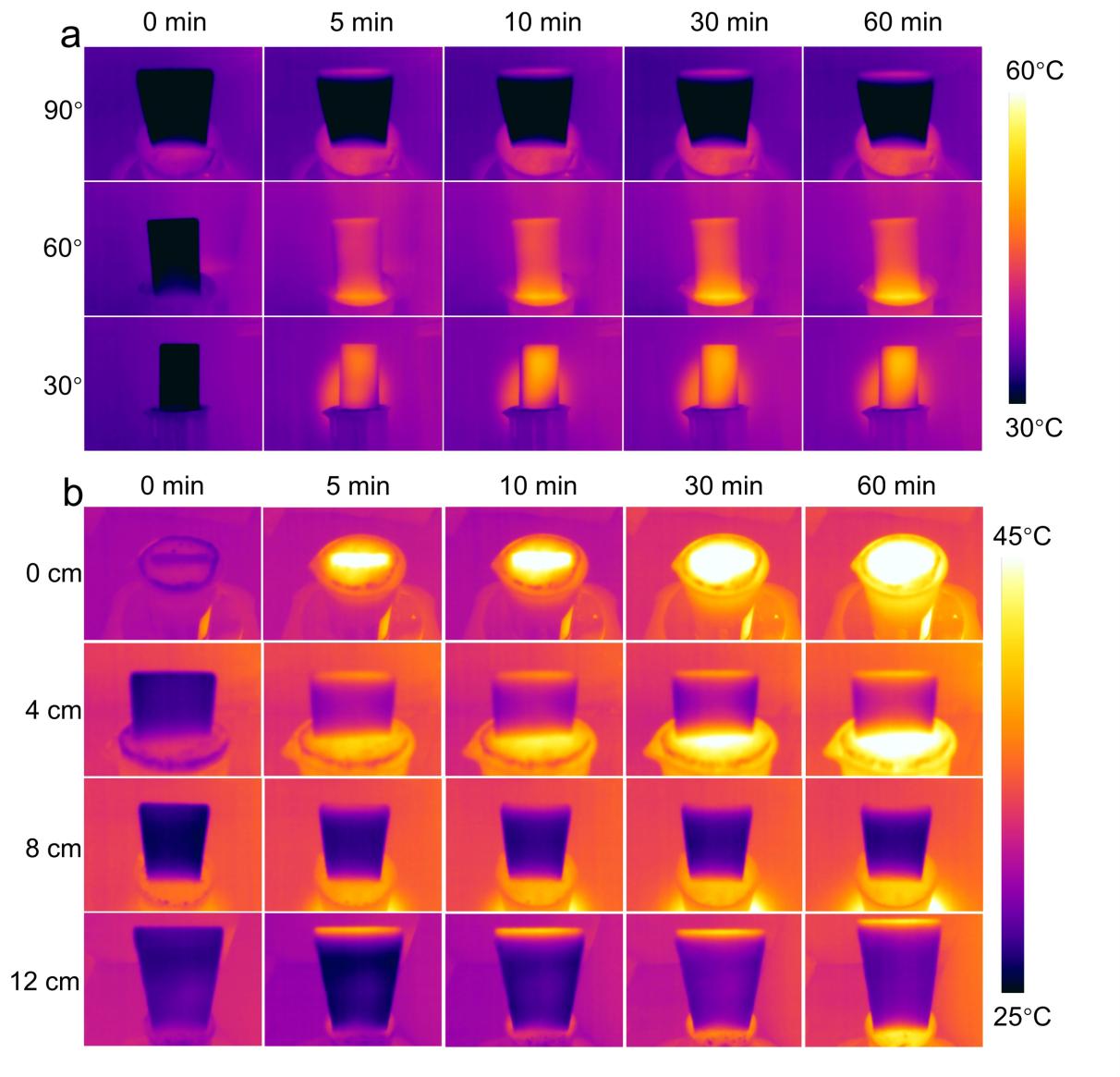


**Fig. S7** IR images of the NF-WA at (**a**) different illumination angles and (**b**) heights of the evaporator under solar intensity of 1 kW m^-2^


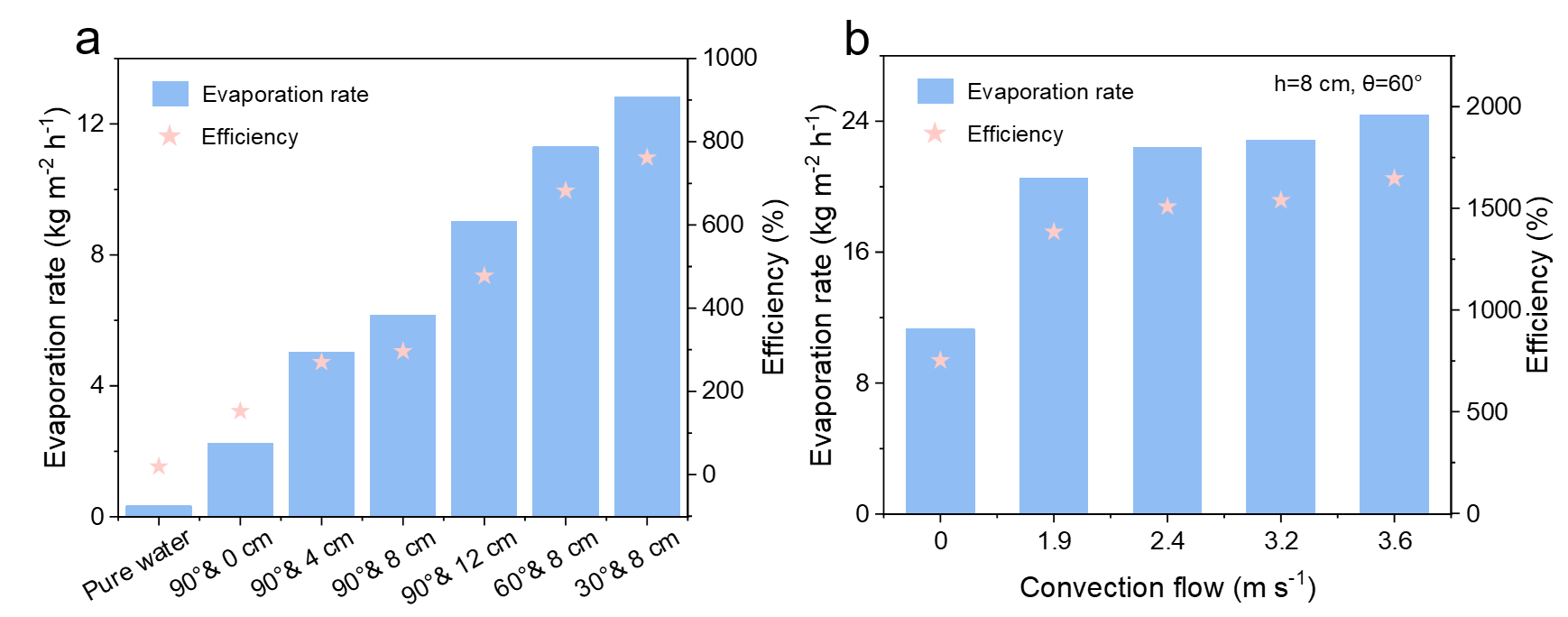


**Fig. S8** (**a**) Evaporator rate and efficiency of pure water and different heights of NF-WA with 90°, 60° and 30° solar altitudinal angels under 1.0 sun irradiation. (**b**) Evaporator rate and efficiency of 8 cm NF-WA with different air convection flow under 1.0 sun irradiation

**Fig. S9** A comparison of the evaporation rate of the NF-WA against those of the reported solar water purifiers. The details are shown in Table S2


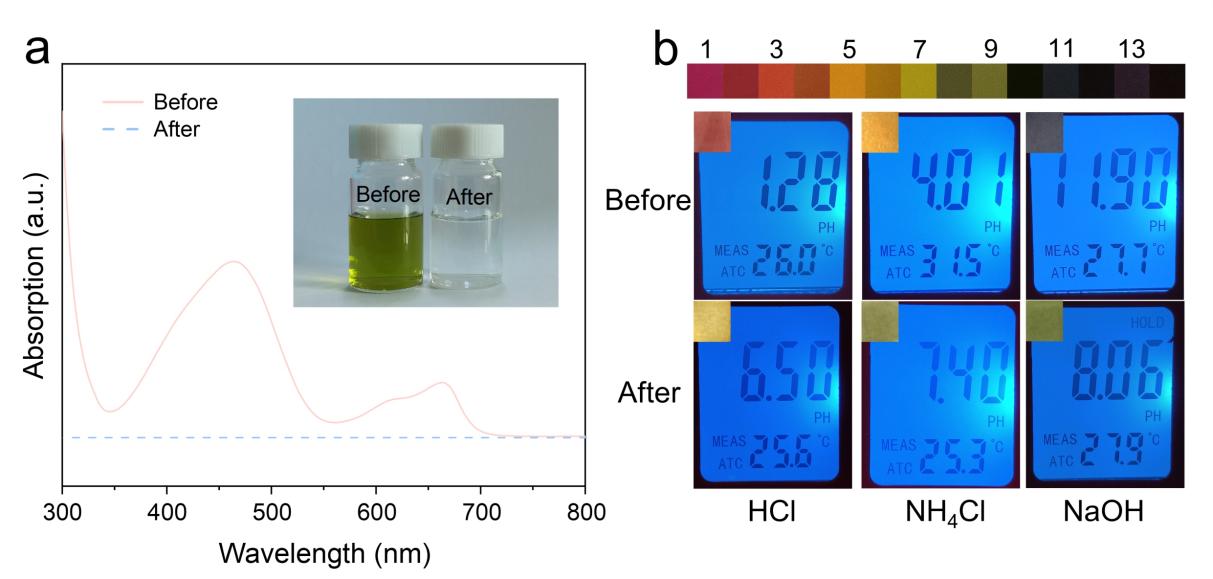


**Fig. S10** (**a**) UV-Vis absorption spectra of the mixture of methyl orange (MO) and methylene blue (MB) solution and corresponding after purified water. (**b**) Changes of pH in HCl, NH_4_Cl and NaOH solutions before and after evaporation


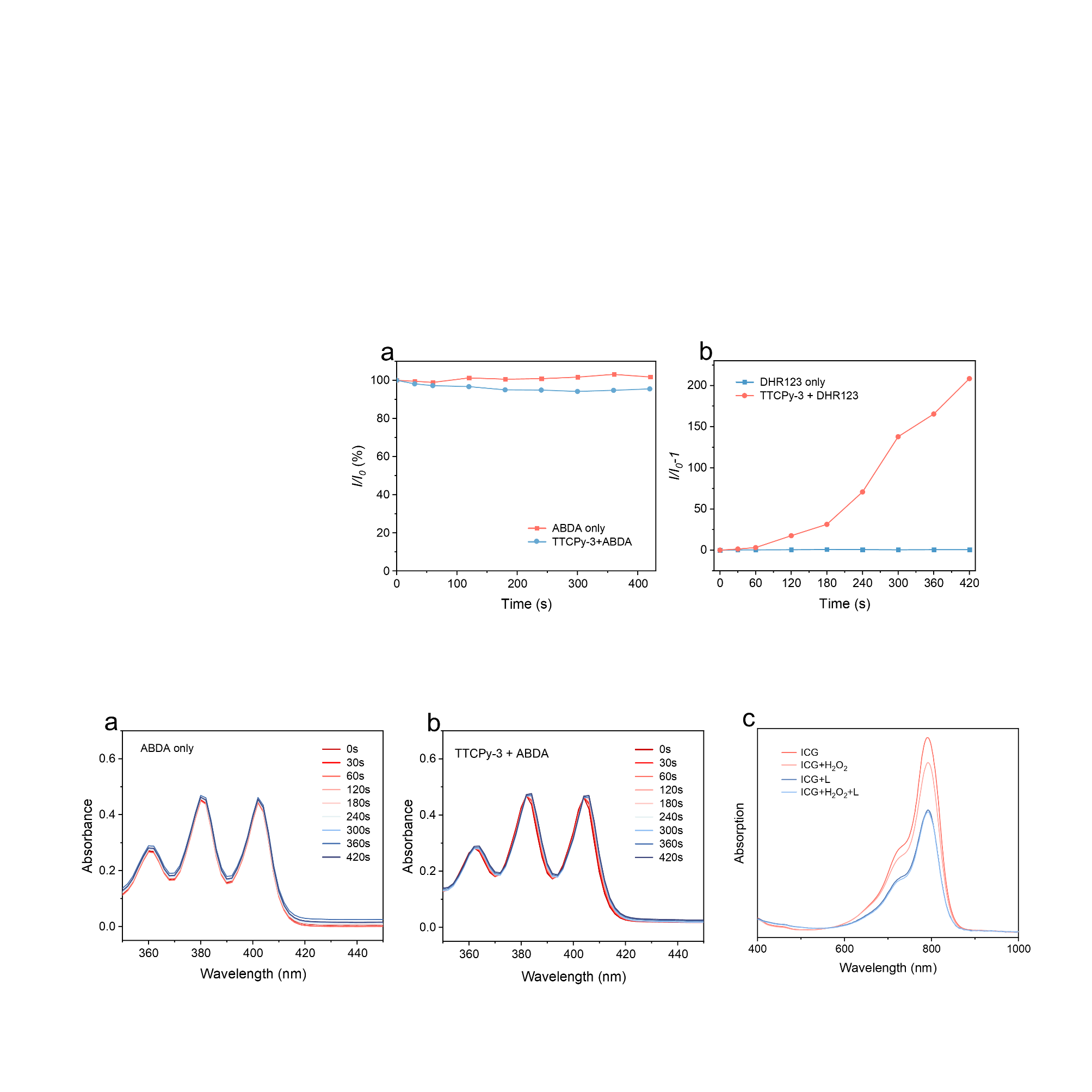


**Fig. S11** ROS generation of TTCPy-3 upon white light irradiation: (**a**) decomposition rates of ABDA for ^1^O_2_ detection and (**b**) DHR 123 for O_2_^−•^ in the presence of TTCPy-3 (1 μM) upon white light irradiation (30 mW cm^-2^) for different time


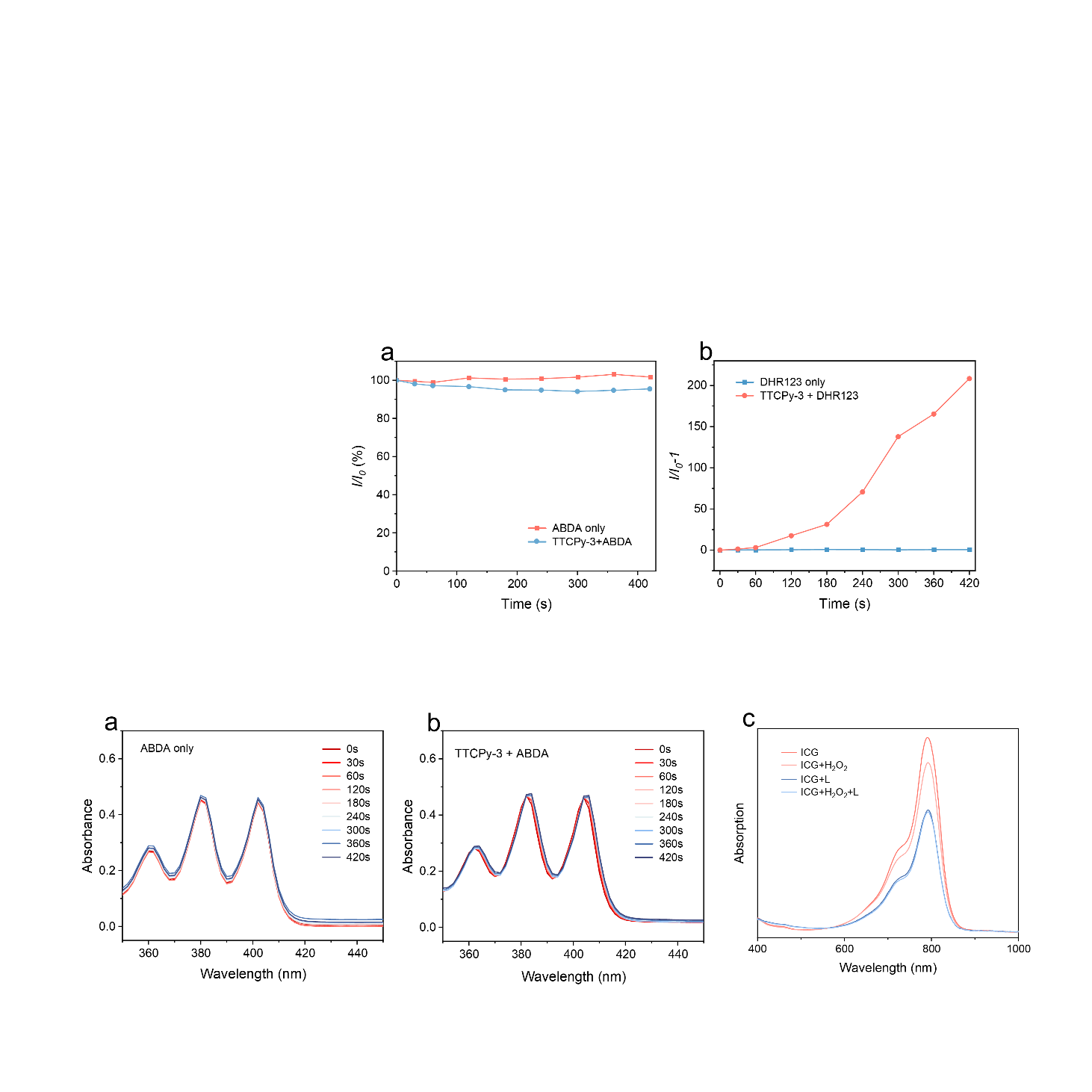


**Fig. S12** (**a**) UV-vis spectra of ABDA in the absence of TTCPy-3 under white light irradiation in DMSO/water (v:v) = 1/100. (**b**) UV-vis spectra of ABDA in the presence of TTCPy-3 under white light irradiation in DMSO/water (v:v) = 1/100. [TTCPy-3] = 1×10^-6^ M, [ABDA] = 5×10^-5^ M, time interval for recording the UV-vis spectra: 60 s, Light power: 30 mW cm^-2^. (**c**) The absorption spectra of ICG in DMSO solution with/without light irradiation for 24 h, and with/without the addition of 30% H_2_O_2_ solution


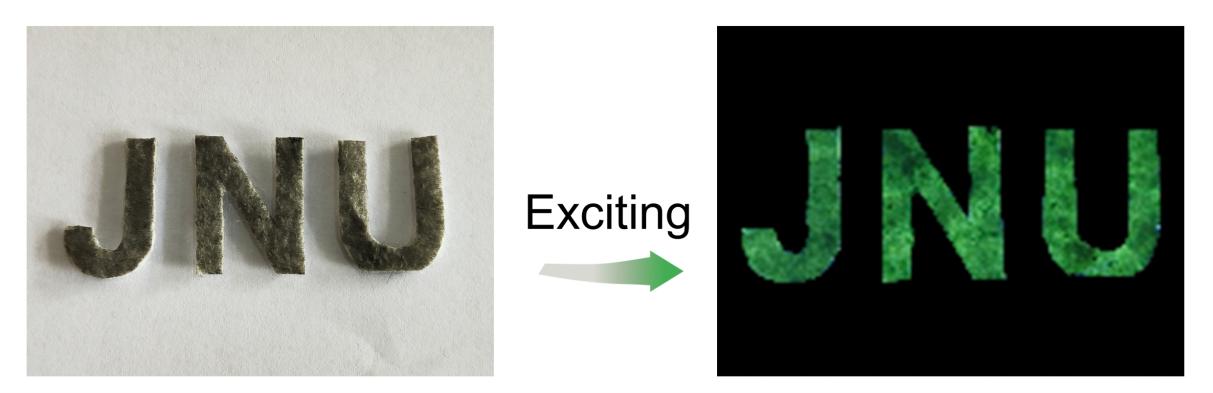


**Fig. S13** Naked eye fluorescent photos of AIE-dopped nonwoven fabric under 365 nm UV light illumination


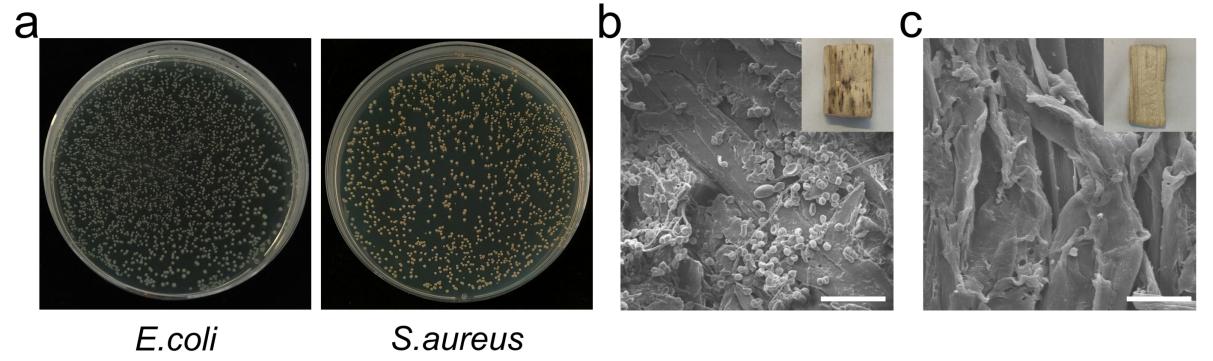


**Fig. S14** (**a**) The bacterial inactivation performance of NF-WA evaporator without TTCPy-3 was assessed under 1.0 sun irradiation for 30 minutes. (**b**) Morphologies of the natural balsa wood surface after 72 h floating on the water. (**c**) Morphologies of wood aerogel surface of NF-WA after 72 h floating on the water. Mildew was observed on the surface. Scale bar: 20 μm


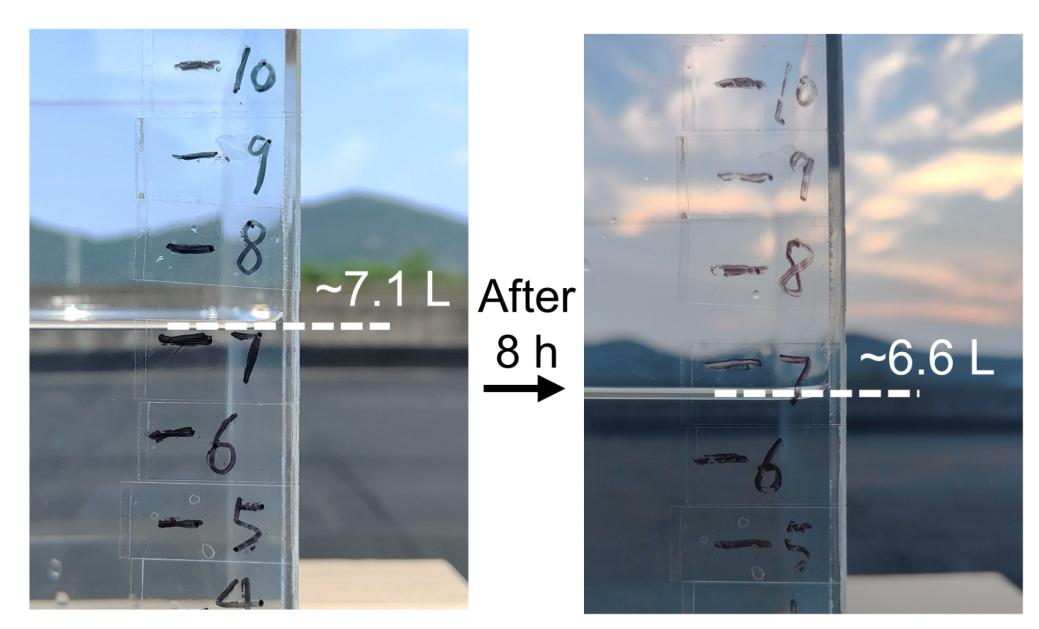


**Fig. S15** The volume change of simulated wastewater evaporated by NF-WA array exposed to natural light for 8 h on July 24, 2023

**Table S1** Size parameter of NF-WA for solar evaporator construction

| Evaporator height | Nonwoven fabric | Wood aerogel | Area of top surface |
| --- | --- | --- | --- |
| 4 cm NF-WA | 9 cm×0.9 cm | 4 cm×0.9 cm×4 cm | 3.6 cm^2^ |
| 8 cm NF-WA | 17 cm×0.9 cm | 4 cm×0.9 cm×8 cm | 3.6 cm^2^ |
| 12 cm NF-WA | 25 cm×0.9 cm | 4 cm×0.9 cm×12 cm | 3.6 cm^2^ |

**Table S2** Comparison of evaporation rates and evaporation efficiencies of various solar wastewater evaporator under 1.0 sun irradiation (W: wood; F: fabric; A: aerogel; H: hydrogel).

| No. | Evaporation rate (kg m^-2^ h^-1^) | Efficiency (%) | Types | Dimension | Anti-biofouling | Refs. |
| --- | --- | --- | --- | --- | --- | --- |
| 1 | 0.64 | 57 | W, F | 2D | No | [S[1](#_ENREF_1)] |
| 2 | 1.31 | 91.5 | W | 2D | No | [S[2](#_ENREF_2)] |
| 3 | 1.45 | 91.5 | F | 2D | Yes | [S[3](#_ENREF_3)] |
| 4 | 1.47 | 91 | H | 2D | Yes | [S[4](#_ENREF_4)] |
| 5 | 1.65 | 95 | H, F | 2D | No | [S[5](#_ENREF_5)] |
| 6 | 1.92 | 90.7 | W | 3D | No | [S[6](#_ENREF_6)] |
| 7 | 1.95 | 116 | F | 3D | No | [S[7](#_ENREF_7)] |
| 8 | 2.03 | 139.4 | A | 3D | No | [S[8](#_ENREF_8)] |
| 9 | 2.05 | 97.7 | W | 3D | Yes | [S[9](#_ENREF_9)] |
| 10 | 2.42 | / | F | 2D | No | [S[10](#_ENREF_10)] |
| 11 | 2.5 | 95 | H | 3D | No | [S[11](#_ENREF_11)] |
| 12 | 2.58 | / | W | 3D | No | [S[12](#_ENREF_12)] |
| 13 | 2.75 | 167 | F | 3D | No | [S[13](#_ENREF_13)] |
| 14 | 2.75 | 82 | W | 3D | No | [S[14](#_ENREF_14)] |
| 15 | 2.9 | 80 | W | 3D | No | [S[15](#_ENREF_15)] |
| 16 | 3.06 | 65.5% | H | 3D | Yes | [S[16](#_ENREF_16)] |
| 17 | 3.06 | 94.5 | F | 2D | No | [S[17](#_ENREF_17)] |
| 18 | 3.17 | / | A | 3D | No | [S[18](#_ENREF_18)] |
| 19 | 3.2 | 90 | H | 2D | No | [S[19](#_ENREF_19)] |
| 20 | 3.4 | 91 | H | 2D | Yes | [S[20](#_ENREF_20)] |
| 21 | 3.6 | / | F | 3D | Yes | [S[21](#_ENREF_21)] |
| 22 | 3.74 | 192.7 | H, F | 3D | No | [S[5](#_ENREF_5)] |
| 23 | 3.91 | / | W | 3D | No | [S[22](#_ENREF_22)] |
| 24 | 1.94 | / | A | 3D | No | [S[23](#_ENREF_23)] |
| 25 | 2.39 | 93.7 | A | 3D | No | [S[24](#_ENREF_24)] |
| 26 | 3.6 | 189 | A | 3D | Yes | [S[25](#_ENREF_25)] |
| 24 | 6.16 | 296.25 | W, F | 3D | Yes | Ours |

**Supplementary References**

1. G. Ni, S.H. Zandavi, S.M. Javid, S.V. Boriskina, T.A. Cooper et al., A salt-rejecting floating solar still for low-cost desalination. Energy Environ. Sci. **11**, 1510–1519 (2018). <https://doi.org/10.1039/c8ee00220g>
2. C. Dang, H. Wang, Y. Cao, J. Shen, J. Zhang et al., Ultra salt-resistant solar desalination system *via* large-scale easy assembly of microstructural units. Energy Environ. Sci. **15**, 5405–5414 (2022). <https://doi.org/10.1039/d2ee03341k>
3. X. Guan, P. Kumar, Z. Li, T.K.A. Tran, S. Chahal et al., Borophene embedded cellulose paper for enhanced photothermal water evaporation and prompt bacterial killing. Adv. Sci. **10**, e2205809 (2023). <https://doi.org/10.1002/advs.202205809>
4. Y. Lei, J. Wang, B. Jiang, H. Liu, M. Ding et al., Revolutionary solar evaporation system: Harnessing the power of bacterial cellulose/Ag NPs/polypyrrole with its promoted antibacterial applications. Appl. Surf. Sci. **644**, 158751 (2024). <https://doi.org/10.1016/j.apsusc.2023.158751>
5. L. Han, H. Zhou, M. Fu, J. Li, H. Ma et al., Manufacturing robust MXene-based hydrogel-coated cotton fabric *via* electron-beam irradiation for efficient interfacial solar evaporation. Chem. Eng. J. **473**, 145337 (2023). <https://doi.org/10.1016/j.cej.2023.145337>
6. D. Fan, Y. Lu, X. Xu, Y. Tang, H. Zhang et al., Multifunctional wood-based hydrogels for wastewater treatment and interfacial solar steam generation. Chem. Eng. J. **471**, 144421 (2023). <https://doi.org/10.1016/j.cej.2023.144421>
7. [7] Q. Zhang, R. Hu, Y. Chen, X. Xiao, G. Zhao et al., Banyan-inspired hierarchical evaporators for efficient solar photothermal conversion. Appl. Energy **276**, 115545 (2020). <https://doi.org/10.1016/j.apenergy.2020.115545>
8. K. Zhou, L. Yin, K. Gong, Q. Wu, 3D Vascular-structured Flame-retardant Cellulose-based photothermal aerogel for Solar-driven interfacial evaporation and wastewater purification. Chem. Eng. J. **464**, 142616 (2023). <https://doi.org/10.1016/j.cej.2023.142616>
9. J. Wang, Z. Chen, L. Feng, F. Yu, C. Ran et al., Plants transpiration-inspired antibacterial evaporator with multiscale structure and low vaporization enthalpy for solar steam generation. Nano Energy **114**, 108631 (2023). <https://doi.org/10.1016/j.nanoen.2023.108631>
10. S. Zhou, Z. Qiu, M. Strømme, C. Xu, Solar-driven ionic power generation *via* a film of nanocellulose @ conductive metal–organic framework. Energy Environ. Sci. **14**, 900–905(2021). <https://doi.org/10.1039/d0ee02730h>
11. X. Zhou, F. Zhao, Y. Guo, Y. Zhang, G. Yu, A hydrogel-based antifouling solar evaporator for highly efficient water desalination. Energy Environ. Sci. **11**, 1985–1992(2018). <https://doi.org/10.1039/c8ee00567b>
12. Y. Li, Y. Ma, Y. Liao, L. Ji, R. Zhao et al., High-entropy-alloy-nanoparticles enabled wood evaporator for efficient photothermal conversion and sustainable solar desalination. Adv. Energy Mater. **12**, 2203057 (2022). <https://doi.org/10.1002/aenm.202203057>
13. Y. Zhao, D. You, Y. Chen, Q. Pan, Z. Su et al., Highly efficient 3D evaporator for interfacial solar steam generation and wastewater treatment. Energy Technol. **12**, 2300586 (2024). <https://doi.org/10.1002/ente.202300586>
14. K. Sheng, M. Tian, J. Zhu, Y. Zhang, B. Van der Bruggen, When coordination polymers meet wood: from molecular design toward sustainable solar desalination. ACS Nano **17**, 15482–15491 (2023). <https://doi.org/10.1021/acsnano.3c01421>
15. Q.-F. Guan, Z.-M. Han, Z.-C. Ling, H.-B. Yang, S.-H. Yu, Sustainable wood-based hierarchical solar steam generator: a biomimetic design with reduced vaporization enthalpy of water. Nano Lett. **20**, 5699–5704 (2020). <https://doi.org/10.1021/acs.nanolett.0c01088>
16. Z. Wang, R. Jin, S. Zhang, X. Han, P. Guo et al., Bioinspired, sustainable, high-efficiency solar evaporators for sewage purification. Adv. Funct. Mater. **33**, 2306806 (2023). <https://doi.org/10.1002/adfm.202306806>
17. R. Zheng, T. Lin, W.-L. Zhao, R. Yin, H. Li et al., Hierarchical CoMn-LDH based photothermal membrane with low evaporation enthalpy and narrow bandgap toward highly efficient Solar-Driven evaporation. Chem. Eng. J. **470**, 144103 (2023). <https://doi.org/10.1016/j.cej.2023.144103>
18. J. Sun, R. Teng, J. Tan, M. Xu, C. Ma et al., An integrated cellulose aerogel evaporator with improved thermal management and reduced enthalpy of evaporation using a hierarchical coordinated control strategy. J. Mater. Chem. A **11**, 6248–6257 (2023). <https://doi.org/10.1039/d2ta07122c>
19. Y. Guo, H. Lu, F. Zhao, X. Zhou, W. Shi et al., Biomass-derived hybrid hydrogel evaporators for cost-effective solar water purification. Adv. Mater. **32**, e1907061 (2020). <https://doi.org/10.1002/adma.201907061>
20. Y. Guo, C.M. Dundas, X. Zhou, K.P. Johnston, G. Yu, Molecular engineering of hydrogels for rapid water disinfection and sustainable solar vapor generation. Adv. Mater. **33**, e2102994 (2021). <https://doi.org/10.1002/adma.202102994>
21. H. Li, W. Zhu, M. Li, Y. Li, R.T.K. Kwok et al., Side area-assisted 3D evaporator with antibiofouling function for ultra-efficient solar steam generation. Adv. Mater. **33**, e2102258 (2021). <https://doi.org/10.1002/adma.202102258>
22. J. Tang, T. Zheng, Z. Song, Y. Shao, N. Li et al., Realization of low latent heat of a solar evaporator *via* regulating the water state in wood channels. ACS Appl. Mater. Interfaces **12**, 18504–18511 (2020). <https://doi.org/10.1021/acsami.0c01261>
23. X. Zhao, H. Zhang, K.-Y. Chan, X. Huang, Y. Yang et al., Tree-inspired structurally graded aerogel with synergistic water, salt, and thermal transport for high-salinity solar-powered evaporation. Nano-Micro Lett. **16**, 222 (2024). <https://doi.org/10.1007/s40820-024-01448-8>
24. Q. Chen, M. Choi, H. Chen, J. Kim, C. Qin et al., Tree-inspired aerogel comprising nonoxidized graphene flakes and cellulose as solar absorber for efficient water generation. Nano Lett. **24**, 10583–10591 (2024). <https://doi.org/10.1021/acs.nanolett.4c02742>
25. T. Wang, M. Li, H. Xu, X. Wang, M. Jia et al., MXene sediment-based poly(vinyl alcohol)/sodium alginate aerogel evaporator with vertically aligned channels for highly efficient solar steam generation. Nano-Micro Lett. **16**, 220(2024). <https://doi.org/10.1007/s40820-024-01433-1>
